# Diversity of wild edible plants of Guinea-Bissau (West Africa): traditional uses and trade

**DOI:** 10.1186/s13002-025-00825-w

**Published:** 2025-12-24

**Authors:** Bucar Indjai, Amélia Frazão-Moreira, Pedro Segurado, Maria Manuel Romeiras, Luís Catarino

**Affiliations:** 1 CEF—Forest Research Centre, School of Agriculture, University of Lisbon, Tapada da Ajuda, 1349-017 Lisbon, Portugal; 2 LEAF—Linking Landscape, Environment, Agriculture and Food, School of Agriculture, University of Lisbon, Tapada da Ajuda, 1349-017 Lisbon, Portugal; 3 INEP—Instituto Nacional de Estudos e Pesquisa, Avenida Dos Combatentes da Liberdade da Pátria, Complexo Escolar 14 de Novembro, Caixa Postal 112 Bissau, Guinea-Bissau; 4 CE3C—Centre for Ecology, Evolution and Environmental Changes & CHANGE—Global Change and Sustainability Institute, Faculty of Sciences, University of Lisbon, Campo Grande, 1749-016 Lisboa, Portugal; 5 CRIA - Centre for Research in Anthropology, School of Social Sciences and Humanities, NOVA University of Lisbon (NOVA FCSH) and IN2PAST Associate Laboratory, Av. de Berna, 26 C, 1069-061 Lisbon, Portugal; 6 TERRA Associate Laboratory, School of Agriculture, University of Lisbon, Tapada da Ajuda, 1349-017 Lisbon, Portugal

**Keywords:** Ethnobotany, Useful plants, Livelihoods, Non-timber forest products, West Africa, Food security

## Abstract

**Background:**

Wild edible plants (WEPs) are among the most important non-timber forest products harvested because of their contribution for food security of local populations and generation of income for families. To evaluate the importance of WEPs in Guinea-Bissau (West Africa), this study characterized the diversity of their uses in the country and discusses their current socioeconomic relevance and potential for sustainable use, and conservation.

**Methods:**

Data on WEPs were collected during fieldwork and market surveys carried out across the country, as well as from bibliographic and herbarium sources. A total of 62 interviews (49 women and 13 men, aged 15–60 years) were conducted between November 2021 and August 2024. A set of variables concerning the collection, trade, and consumption of WEPs was drawn up to classify the socioeconomic importance of the species traded.

**Results:**

We documented 115 WEPs from 45 families and 89 genera; 111 of them are native species and four are introduced and naturalized in Guinea-Bissau. Most of the WEP are woody plants found in woodlands and savannah woodlands. Fruits, followed by leaves and underground organs are the most usually consumed parts, mainly eaten raw. Thirty-nine WEPs are traded in the markets, eight of which can be considered as having high socioeconomic importance both as food and income source. Some patterns of use can be highlighted: children consume a larger number of wild fruits than adults, certain plants are only eaten in periods of food shortage, and several species are highly valued in the markets.

**Conclusions:**

In Guinea-Bissau, WEPs play a key role in the traditional diet of local communities, especially when crops are scarce, thus ensuring food security, particularly for the most vulnerable populations. The sustainable use of WEPs can contribute to the well-being of local populations and to the conservation of the natural resources and ecosystems in this West African country.

**Supplementary Information:**

The online version contains supplementary material available at 10.1186/s13002-025-00825-w.

## Introduction

Wild edible plants (WEPs), defined as non-cultivated and non-domesticated edible plants, are vital to enhance food security and generate income in most of the tropical countries [1, 2]. Harvested from the wild, they are important sources for human nutrition and should not be neglected in terms of food security, good health, and income generation [3, 4].

In sub-Saharan African countries, many rural communities still collect, consume, and commercialize wild food plants [5]. In diets with little variety of species, the consumption of these plants is particularly vital in times of food scarcity and adopted as a survival strategy and to support the family economy [6]. Several WEPs have acquired economic importance, even in global trade, such as baobab (*Adansonia digitata* L.) [7] and amarula (*Sclerocarya birrea* (A.Rich.) Hochst.) [8].

Although the African flora has a large number of edible native species (over 2,000), most of the cultivated plants and some of the wild food plants have been introduced and are mainly of American or Asian origin [9]. Only a small proportion of African native food plants are used, with even fewer being cultivated or sold [10]. However, they can offer a viable alternative to imported food products and crops [11]. Moreover, local foods play a crucial role for rural African families, and their sustainable use can help protect fragile ecosystems [12]. In recent years, there have been remarkable advances in the digitization and accessibility of data on Africa’s plant diversity, with databases offering detailed information on the traditional uses [13], as well as details on their ecology and distribution of species [14], thus providing valuable platforms for researchers, conservationists, and policymakers.

In West Africa, many plant species fulfill the basic needs of rural populations, who use them for several purposes (fibers, phytochemicals, building materials, artifacts), and to a greater extent, as medicines, food, and/or nutraceuticals [15, 16]. There are differences in consumption, depending on ethnicity, culture, gender, and age class. Even in the same country, many plants prized by some communities are not used at all or not appreciated by others, often for cultural or religious reasons [e.g., 17, 18]. In some cases, this can explain the ignorance about the uses and properties of local plant resources [19]. Nevertheless, traditional knowledge of the uses and properties of WEPs is a valuable cultural asset for local communities, though it is sometimes threatened by cultural erosion [20, 21].

In Guinea-Bissau, a small West African country, several kinds of edible consumption of wild plants can be considered [22]. The leaves and flowers are used as vegetables in stews, either dried and ground or fresh. A large diversity of fruits is eaten locally, and many are also traded in the city markets and some even exported. Some wild edible roots and tubers are appreciated, but most of them are used mainly in times of scarcity as resource food. The knowledge about the diversity and properties of the Guinea-Bissau useful plants is the basis for their valorization, and in some cases, to develop processes of domestication of promising plants [23]. However, despite their importance as food sources and primary therapeutic strategy for many communities, the degradation of vegetation of different ecosystems and the erosion of ethnobotanical knowledge are jeopardizing the preservation and valorization of these plant genetic resources in Guinea-Bissau [21, 24].

Although several works have been done on local knowledge of wild plant uses in Guinea-Bissau [e.g., 20, 23–29], there is still limited documented information about WEPs for the country. Some studies already highlighted their importance, both as valuable natural resources and as complementary food sources, as well as a source of additional income for many families in the country [23]. These products are commonly used in local diets and are often sold along roadsides by collectors, mainly women and young people, in local weekly markets (*lumus* in Guinea-Bissau Creole) and in urban markets [30, 31]. There is also trade with neighboring countries, namely, Senegal and The Gambia, or sold in European and American countries to meet the needs of immigrant Guinean communities, such as *A. digitata, Dialium guineense* Willd.,* Parkia biglobosa* (Jacq.) R.Br. ex G.Don*,* and *Saba senegalensis* (A.DC.) Pichon.

The main objective of this work was to ascertain how important WEPs are in Guinea-Bissau, by identifying and characterizing them, and to discuss their current socioeconomic relevance and potential for sustainable use to fight food insecurity in the country. For these purposes, information on the plant species and the local traditional knowledge about them was assessed. This information can support the preservation of traditional knowledge, providing valuable tools for decision-makers and planners to design effective conservation and development practices, and promote the sustainable use of natural resources, especially of non-wood forest products. Our results will raise awareness on the need for the sustainability, preservation of traditional ecological knowledge, and the socioeconomic importance of WEPs in Guinea-Bissau.

## Methods

### Description of the study area

**Fig. 1:**
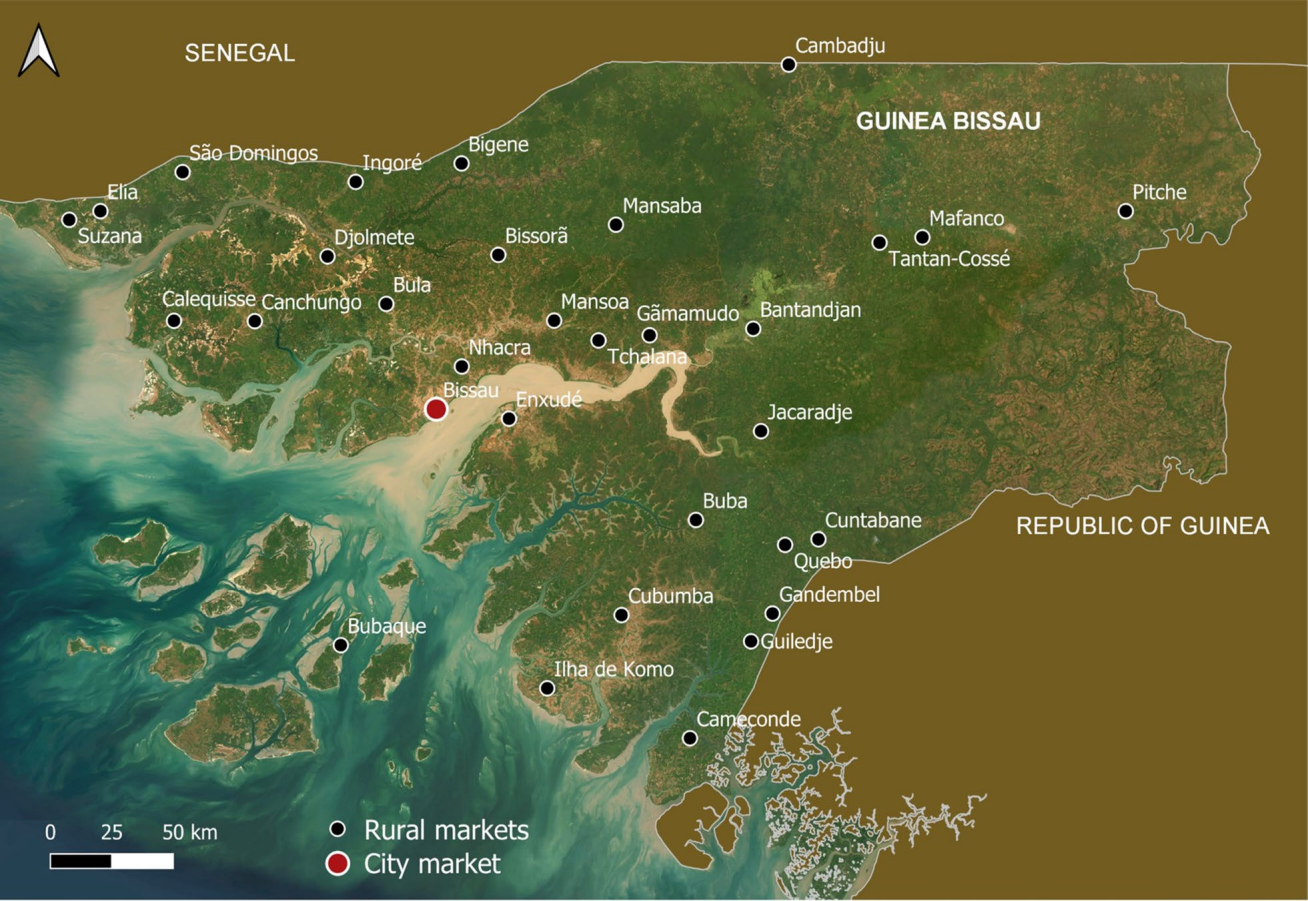
Map of Guinea-Bissau with the location of the local and city markets where data was collected from. The coordinates of the 32 visited markets and brief characterization of their locations are presented in Table S1

This study was conducted in Guinea-Bissau (Fig. 1), a small West African country hosting more than two million people in an area of 36,125 km^2^ [22]. Outside Bissau, the capital, the population is mainly rural and very few services and infrastructures are available. The climate is tropical, with alternating wet and dry seasons, and the vegetation can be classified according to three main zones: (i) littoral (mangroves, palm groves, woodlands, and forest); (ii) transitional (mosaic of woodland and savanna woodland; tall grass savanna in the inner valleys); and (iii) hinterland (woodland, savanna woodland, and shrubby or herbaceous steppes) [24]. Due to slash-and-burn agriculture, secondary vegetation dominates in Guinea-Bissau [32].

Although small, the country comprises a large ethnocultural diversity, with about 30 ethnolinguistic groups mainly Muslims or following traditional African religions, and to a smaller extent, Christians. The 2009 population census (the most recent one with ethnic data) indicates that the largest groups were the Fula (Fulani), Balanta, Mandinga (Mandinka), Pepel, and Manjaco (Manjak) [22]. A more recent estimate suggests that 30% of the country’s population is Balanta, 30% Fula (Fulani), 14% Manjaco (Manjak), 13% Mandinga (Mandinka), 7% Pepel, and 6% from smaller groups such as Beafada, Bijagó, Felupe, Nalu, and Sosso (Sussu) [33]. Guinea-Bissau’s official language is Portuguese, but Guinea-Bissau Creole and the ethnic languages are those most spoken [21].

The main staple food in the country is rice, grown in lowland rainfed systems, in mangrove rice fields and in upland rainfed cropping systems (shifting agriculture). Traditionally, some ethnolinguistic groups living in coastal areas (e.g., Balata, Felupe, and Pepel) specialize in mangrove rice cultivation, while others who live mainly inland (e.g., Fula, Mandinga, Manjaco), cultivate rice using the slash-and-burn system. Floodplain rice is practiced across the country by most groups. Other important foods include cassava, sweet potatoes, peanuts, beans, millet, maize, and sorghum, as well as fish and shellfish in coastal areas, and game and animal husbandry inland. Over the last decades, a boom of cashew plantation has been occurring in the country. This phenomenon and population growth leads to the conversion of forests into agricultural land and cashew plantations [34].

### Data collection

The data on the availability, harvesting, processing, preservation, trade, and food properties locally reported for the WEPs in Guinea-Bissau were obtained from three main sources: 1) ancillary data from herbarium vouchers housed at LISC herbarium, University of Lisbon; 2) published works and reports resulting from the authors’ work in Guinea-Bissau since the 2000 decade (e.g., Catarino et al. [21, 23–25]; Indjai et al. [26, 27]; Indjai [28]; Indjai & Catarino [29]); and 3) recent in-depth market surveys by the first author.

The market surveys were carried out from November 2021 to January 2022, in February 2023, and from March to August 2024; one city market (Bandim, the largest market in Bissau) and 31 local countryside informal markets (*lumus*), distributed across the whole country were visited (Fig. 1; Table S1) to collect data on the WEP products traded over the year. Direct observation was employed as the initial market survey to identify and record WEPs available for sale (see market questionnaires in Table S2). This procedure was conducted stall by stall in each market, allowing for systematic documentation of species diversity and follows the methodological framework proposed by Albuquerque et al. [35]. Sixty-two vendors were interviewed: 49 women and 13 men, aged between 15 and 60 years (average: 37), selected by purposive sampling based on availability and knowledge of forest food products. The methodological approach took into account time constraints and the dynamic nature of market environments, particularly in the *lumus*, where commercial activity is fast-paced and informal. The interviews were semi-structured and conducted shortly after the study presentation and verbal informed consent was obtained, in accordance with the ethical standards for ethnobotanical research in market contexts, as described by Albuquerque et al. [35] (Table S2). This approach was complemented by direct and detailed observations of interactions between people in the *lumu*, allowing for a deeper understanding of the social dynamics, knowledge exchange, and cultural practices associated to WEPs. Their occupations varied between market vendors (*bideiras*, a local term for women who sell products in fixed stalls or on the street) (28), farmers (19), public servants (9), students (3), and housekeepers (3). The participants represented 15 ethnic groups, distributed as follows: Balanta (18), Manjaco (9), Beafada (7), Mandinga (6), Pepel (5), Fula (3), Nalu (3), Sosso (3), Mancanha (2), Balanta Mané, Banhum, Cassanga, Felupe, Saracolé, and Tanda (1 each). Participants were classified according to their relationship with WEPs: as sellers (29), collector-sellers (16), consumers (12), and collectors (5).

Plants collected during the surveys were identified by the first and last authors and subsequently deposited in the LISC herbarium. A voucher for each species is provided in Table S3. To complement data from the field and market surveys with information concerning other uses, online databases were consulted [13, 14] and a comprehensive review of the literature was made [15, 16, 20, 23, 25–27, 30, 31, 36–50]. The plant names were checked and updated using online taxonomical databases [14, 51].

To analyze the data, an index was designed based on a set of variables related with the collection, trade, and consumption of WEPs: Availability; Harvesting and Processing; Preservation; Commercialization; Food properties; Other uses (Table 1). An importance value was assigned to each variable, ranging from 1 (least important) to 4 (most important), and the total score was used as an index to classify the socioeconomic importance of the traded species (Table 2).

**Table 1:** Variables and scores used to estimate the socioeconomic importance of marketed species

Variable\Score	1	2	3	4
Availability	Difficult to obtain	Territorially or habitat restricted	Restricted in time and space	Easy to obtain for most of the year and in quantity
Harvesting and Processing	Difficult to harvest and process	Difficult to process	Difficult to harvest	Easy harvesting and processing
Preservation	Quick deterioration	Preserved only for short periods (< 1 week)	Kept for long periods (< 1 month)	Easy storage for long periods
Commercialization	Marketed sporadically or only locally	Good market acceptance; seasonally marketed	Widely accepted on the market; sold most of the year	Marketed locally, nationally, and internationally, widely accepted
Food properties	Referred locally as food	Referred locally as having good eating properties	Good properties reported locally and/or in at least one published work	Good properties reported locally and on various published works
Other uses	No other known uses	With one other known use	With two or more other uses	With several other uses

**Table 2:** Wild edible plants (WEPs) traded in Guinea-Bissau according to the 2021–2024 surveys, and their herbarium voucher and vernacular name in Guinea-Bissau Creole

Species	Voucher	Vernacular name in Guinea-Bissau Creole	Socioeconomic variables						
			Availability	Harvest and Processing	Preservation	Trade	Properties	Other uses	Socioeconomic importance
**Amaranthaceae**									
*Amaranthus cruentus* L	LISC131171	brêdo-fêmea, djambô, bordor	2	4	1	1	1	3	**12**
*Amaranthus spinosus* L	LISC131172	brêdo, bride	2	4	1	1	1	3	**12**
*Amaranthus viridis* L	LISC131173	brêdo, borbor	2	4	1	1	3	3	**14**
**Anacardiaceae**									
*Spondias mombin* L	LISC003056	mandiple	3	4	1	3	4	4	**19**
**Anisophylleaceae**									
*Anisophyllea laurina* R.Br. ex Sabine	LISC003008	miséria, pau-miséria, pó-de-miséria, po-miséria	3	4	1	3	4	4	**19**
**Annonaceae**									
*Uvaria chamae* P.Beauv	LISC093178	banana-de-santcho, banana-di-macacou	2	3	1	2	3	4	**15**
*Xylopia aethiopica* (Dunal) A.Rich	LISC093260	malagueta-preta-de-Guiné, malagueta-di-mato	2	3	3	3	4	4	**19**
**Apocynaceae**									
*Landolphia dulcis* (Sabine ex G.Don) Pichon	LISC131187	cibode, mambimba	3	4	2	1	3	2	**15**
*Landolphia heudelotii* A.DC	LISC131182	fole, fole-macacou, folezinho, mambimba, fole-pequeno	3	4	2	3	4	4	**20**
*Landolphia hirsuta* (Hua) Pichon	LISC131180	fole	2	4	2	1	3	2	**14**
*Landolphia owariensis* P.Beauv	LISC131183	fole-elefante, fole-de-elefante	1	4	3	1	3	4	**16**
*Mondia whitei* (Hook.f.) Skeels	LISC131181	pó-doce	2	4	4	1	3	2	**16**
*Saba comorensis* (Bojer ex A.DC.) Pichon	LISC131179	caba-forô	2	3	3	2	4	2	**16**
*Saba senegalensis* (A.DC.) Pichon	LISC131178	fole, folelifante, fole-de-elefante	4	4	3	4	4	2	**21**
**Arecaceae**									
*Borassus aethiopum* Mart	LISC131188	cibe	2	3	3	1	3	2	**14**
*Borassus akeassii* Bayton, Ouédr. & Guinko	LISC131236	cibe	2	3	3	1	3	2	**14**
*Elaeis guineensis* Jacq	LISC061874	palmeira-de-óleo, palmera, pé-de-tchebém	4	3	4	4	4	4	**23**
**Celastraceae**									
*Salacia senegalensis* (Lam.) DC	LISC120870	mancuba, momboli, mancubaru, mancubar, mesinho-grande	3	4	2	1	3	3	**16**
**Chrysobalanaceae**									
*Neocarya macrophylla* (Sabine) Prance ex F.White	LISC131192	tambacumba, mampatace-gande, tamankumba	3	3	2	1	3	4	**16**
*Parinari excelsa* Sabine	LISC131194	mampatace, mampataz,	3	3	2	1	3	3	**15**
**Combretaceae**									
*Combretum micranthum* G.Don	LISC131195	buco, café, café-bravo, chá-de-buco	3	3	3	1	2	3	**15**
*Terminalia macroptera* Guill. & Perr	LISC131196	karkone, macete, macite	3	3	3	1	2	3	**15**
**Fabaceae**									
*Dialium guineense* Willd	LISC131206	po-de-veludo, pau-veludo	4	3	4	4	4	4	**23**
*Parkia biglobosa* (Jacq.) R.Br. ex G.Don	LISC131207	farôba, farroba, farrobe	4	3	4	4	4	4	**23**
*Piliostigma thonningii* (Schumach.) Milne-Redh	LISC131208	fará, panu-di-kankora	3	3	3	1	3	3	**16**
**Icacinaceae**									
*Icacina oliviformis* (Poir.) J.Raynal	LISC116436	manganasse, manganace	1	4	4	2	2	2	**15**
**Lamiaceae**									
*Vitex doniana* Sweet	LISC131213	acetona-preta, cetona-preta, azeitona-preto	2	3	2	2	2	2	**13**
**Loganiaceae**									
*Strychnos spinosa* Lam	LISC131215	orelha-de-rato	2	2	2	2	2	3	**13**
**Malvaceae**									
*Adansonia digitata* L	LISC102948	cabaceira, cabacera, calabacera	4	3	4	4	4	4	**23**
*Bombax costatum* Pellegr. & Vuillet	LISC102954	polóm-fidalgo, sumauma	3	3	3	3	3	3	**18**
*Ceiba pentandra* (L.) Gaertn	LISC102977	poilão, poilon	4	3	3	3	3	4	**20**
*Cola cordifolia* (Cav.) R.Br	LISC103514	mandjanja	2	2	2	1	3	3	**13**
*Hibiscus cannabinus* L	LISC102432	narcino-branco, baguitche-de-mato	3	4	1	2	2	1	**13**
*Hibiscus surattensis* L	LISC102550	baguitch-di-mato, bajique-do-mato	3	4	1	2	2	1	**13**
**Meliaceae**									
*Carapa procera* DC	LISC112923	cola-amargoso, cola-malegossa	2	4	4	1	2	3	**16**
**Pedaliaceae**									
*Sesamum radiatum* Thonn. ex Hornem	LISC131224	lalo, lalo-caminho	2	1	3	3	3	4	**16**
**Poaceae**									
*Digitaria longiflora* (Retz.) Pers	LISC131226	fundo-bravo	1	2	4	2	2	2	**13**
**Vitaceae**									
*Cissus populnea* Guill. & Perr	LISC124839	canja-di-mato	2	3	1	1	1	1	**10**
**Zingiberaceae**									
*Aframomum alboviolaceum* (Ridl.) K.Schum	LISC131234	belencufa	2	3	2	1	2	3	**13**

Species	Other uses	Type of use	Plant parts used	Preparation	Preservation time	Processing for preservation	Traded product	References
**Amaranthaceae**								
*Amaranthus cruentus* L	Md	Fd	Lv	Bk	Sh	Wp	Lv	[25]
*Amaranthus spinosus* L	Md	Fd	Lv	Bk	Sh	Wp	Lv	[25, 37, 39]
*Amaranthus viridis* L	Md	Fd	Lv	Bk	Sh	Wp	Lv	[20, 25, 37, 39]
**Anacardiaceae**								
*Spondias mombin* L	Md, Bl	Fd, Dr	Fr	Rw, Jc	Sh	Wp	Fr	[16, 20, 25, 27, 30, 36, 40, 42, 45–49]
**Anisophylleaceae**								
*Anisophyllea laurina* R.Br. ex Sabine	Md, Ar, Bd, Or	Fd	Fr	Rw	Sh	Wp	Fr	[15, 20, 25, 40, 46]
**Annonaceae**								
*Uvaria chamae* P.Beauv	Md, Ar, Fb	Fd	Fr	Rw	Sh	Wp	Fr	[15, 16, 20, 25, 27, 31, 40, 43, 45, 46, 49, 50]
*Xylopia aethiopica* (Dunal) A.Rich	Md, Bd, Bl, Bv	Dr, Sp	Fr, Rt	Bk, Mc	Lg	Dr	Fr, Rt	[15, 20, 25, 27, 36, 37, 40, 47, 50]
**Apocynaceae**								
*Landolphia dulcis* (Sabine ex G.Don) Pichon	Md, Ph	Fd	Fr	Rw	Sh	Wp	Fr	[15, 20, 25, 43, 49]
*Landolphia heudelotii* A.DC	Md, Ph, Ar	Fd, Dr	Fr	Rw, Jc	Sh	Wp	Fr, Vn	[15, 16, 20, 25, 30, 40–43, 45–49]
*Landolphia hirsuta* (Hua) Pichon	Md, Ph	Fd	Fr	Rw, Jc	Sh	Wp	Fr	[15, 25]
*Landolphia owariensis* P.Beauv	Md, Ph	Fd, Dr	Fr	Rw, Jc	Sh	Wp	Fr	[25, 49]
*Mondia whitei* (Hook.f.) Skeels	Md, Bl, Bv	Fd, Dr, Sw	Fr, Rt	Rw, Dc, Mc	Vl	Dr	Rt	[15, 25]
*Saba comorensis* (Bojer ex A.DC.) Pichon	Md, Ph	Fd, Dr	Fr	Rw, Jc	Sh	Wp	Fr	[16, 25, 40]
*Saba senegalensis* (A.DC.) Pichon	Md, Bl, Bv	Fd, Dr	Fr	Rw, Jc	Sh	Wp	Fr	[15, 16, 20, 25, 26, 30, 40–46, 48, 49]
**Arecaceae**								
*Borassus aethiopum* Mart	Md, Ar, Bd, Bl, Or, Fb, Bv	Fd, Dr	Fr, Se, Sp	Rw, Bk, Rs	Sh	Dr	Fr, Se, Sp	[15, 16, 25, 37, 38, 40, 41, 48]
*Borassus akeassii* Bayton, Ouédr. & Guinko	Md, Ar, Bd, Bl, Or, Fb, Bv	Fd, Dr	Fr, Se, Sp	Rw, Bk, Rs	Sh	Dr	Fr, Se, Sp	[16, 38, 46, 48, 49]
*Elaeis guineensis* Jacq	Md, Ph, Ar, Bd, Bl, Or, Fb, Bv, Ot	Fd, Dr, Eo	Fr, Se, Sa	Rw, Bk, Rs	Sh, Lg, Vl	Dr	Fr, Eo, Sa	[15, 16, 25, 26, 30, 31, 37, 40, 41, 43, 44, 46, 47, 49]
**Celastraceae**								
*Salacia senegalensis* (Lam.) DC	Md	Fd	Fr	Rw	Sh	Wp	Fr	[15, 20, 25]
**Chrysobalanaceae**								
*Neocarya macrophylla* (Sabine) Prance ex F.White	Md, Ar	Fd	Fr, Se	Rw	Sh	Dr	Fr, Se	[15, 16, 20, 25, 30, 31, 37, 38, 41, 43, 46–48]
*Parinari excelsa* Sabine	Md, Wd, Bv, Ot	Fd	Fr, Se	Rw	Sh	Dr	Fr	[15, 25, 27, 30, 31, 37, 40, 42, 43, 45–47, 49]
**Combretaceae**								
*Combretum micranthum* G.Don	Md, Bd, Bv, Ot	Dr	Lv	Bk	Vl	Dr	Lv	[15, 16, 20, 25, 27, 31, 37, 38, 43, 46]
*Terminalia macroptera* Guill. & Perr	Md	Dr	Lv	Bk	Vl	Dr	Lv	[15, 16, 25, 27, 31, 45, 46, 49]
**Fabaceae**								
*Dialium guineense* Willd	Md, Ar, Bd, Bv	Fd, Dr	Fr, Lv	Rw, Bk	Lg	Dr	Fr	[15, 16, 20, 25, 26, 30, 36, 40, 42, 43, 46–49]
*Parkia biglobosa* (Jacq.) R.Br. ex G.Don	Md, Bv, Ot	Fd, Dr, Sp	Fr, Se	Rw, Ju, Fr	Lg	Dr	Fr, Se	[15, 16, 20, 25, 26, 30, 31, 36–38, 40–43, 46, 47]
*Piliostigma thonningii* (Schumach.) Milne-Redh	Md, Bl, Fb, Bv, Ot	Dr	Lv	Dc	Vl	Dr	Lv	[16, 20, 25, 27, 31, 45]
**Icacinaceae**								
*Icacina oliviformis* (Poir.) J.Raynal	Md	Fd	Rt, Fr	Bk	Vl	Dr	Se	[15, 20, 25, 37, 42]
**Lamiaceae**								
*Vitex doniana* Sweet	Md	Fd	Fr	Rw	Sh	Wp	Fr	[15, 16, 25, 30, 37, 38, 42, 45, 46, 48, 49]
**Loganiaceae**								
*Strychnos spinosa* Lam	Md	Fd	Fr	Rw	Sh	Wp	Fr	[16, 25, 45, 49]
**Malvaceae**								
*Adansonia digitata* L	Md, Ph, Bl, Fb, Bv	Fd, Dr	Fr, Fl, Lv	Rw, Bk, Ju	Vl	Dr	Lv, Fr	[15, 16, 23, 25, 26, 30, 37–43, 46, 48, 49]
*Bombax costatum* Pellegr. & Vuillet	Wd, Bl	Fd	Fl, Lv	Bk	Lg	Dr	Lv, Fl	[15, 16, 23, 25, 31, 39, 45, 49]
*Ceiba pentandra* (L.) Gaertn	Md, Bl, Or	Fd	Lv, Fl	Bk	Vl	Dr	Lv, Fl	[15, 16, 20, 23, 25, 27, 43, 45–47, 49]
*Cola cordifolia* (Cav.) R.Br	Md, Or	Fd	Fr	Rw	Np	Wp	Fr	[15, 16, 25, 43, 45]
*Hibiscus cannabinus* L	Fb	Fd	Lv	Bk	Np	Wp	Lv	[25, 37]
*Hibiscus surattensis* L		Fd	Lv	Bk	Np	Wp	Lv	[15, 25]
**Meliaceae**								
*Carapa procera* DC	Md, Bd, Bl	Fd	Se	Rw	Lg	Dr	Se	[15, 25, 41, 46, 49]
**Pedaliaceae**								
*Sesamum radiatum* Thonn. ex Hornem	Md	Fd	Lv, Fl	Bk	Lg	Dr	Lv, Fl	[15, 20, 23, 25, 37, 39]
**Poaceae**								
*Digitaria longiflora* (Retz.) Pers	Ot	Fd	Se	Bk	Vl	Dr	Se	[15, 25]
**Vitaceae**								
*Cissus populnea* Guill. & Perr		Fd	Fr	Bk	Sh	Wp	Fr	[15, 16, 25]
**Zingiberaceae**								
*Aframomum alboviolaceum* (Ridl.) K.Schum	Md	Fd	Fr	Rw	Sh	Wp	Fr	[15, 25]

Socioeconomic importance (the scores of the variables are explained in Table 1). Additional information: Other uses: *Md* Medicinal, *Ph* Phytochemical, *Ar* Artifacts, *Bd* Building, *Wd* Wood, *Bl* Beliefs, *Or* Ornamental, *Fb* Fibers, *Bv* Beverages, *Ot* Other; Type of use: *Dr* drink, *Fd* food, *Eo* edible oil, *Sp* spice, *Sw* sweetener, Plant parts used: *Fl* flowers, *Fr* fruits, *Lv* leaves, *Rt* roots and underground organs, *Sa* sap, *Se* seeds, *Ss* sprouted seed, Preparation: *Bk* Baked, *Dc* decoction, *Fr* fermented, *Jc* juice, *Mc* maceration in water, *Rw* Raw, *Rs* Roasted, Preservation time: *Sh* short; *Lg* long; *Vl* very long; *Np* not possible, Processing for preservation: *Dr* drying, *Wp* without processing, and Traded product: *Lv* leaves; *Fr* fruits; *Rt* roots and underground organs, *Vn* vinegar; *Sa* sap, *Se* seeds, *Sp* sprouts, *Eo* edible oil

Each variable was assigned an importance score ranging from 1 (least important) to 4 (most important). The sum of these scores was used to construct the index, which served to classify the socioeconomic importance of the traded species (Table 2).

Finally, a comprehensive database on wild edible plants (WEPs) was compiled (Table 2; Table S4), including information on: scientific names, socioeconomic variables, other uses (medicinal, phytochemical, artifacts, building, wood, beliefs, ornamental, fibers, beverages, other), mode of use (drink, food, edible oil, spice, sweetener), plant parts utilized (flowers, fruits, leaves, roots, sap, seeds, sprouts seeds), preparation methods (baked, decoction, fermented, juice, maceration in water, raw, roasted), preservation time (short, long, very long), processing for preservation (drying, without processing), and traded product (leaves, fruits, roots, vinegar, seeds, young shoots, edible oil).

These data were collected in the field by the first author during the market survey campaigns (see above) and checked or completed with published information [e.g., 15; 16; 25; 26; 27].

## Results and discussion

WEPs are collected and consumed across the whole Guinea-Bissau and are sold in three main types of places: by roadsides during the ripening season of each product, in weekly informal markets (locally called *lumus*) in villages and small cities, and in the markets of major cities, such as the Bandim market in Bissau (Fig. 1; [30]). These species are used daily in traditional dishes, eaten by children, as part of special diets, or as famine foods during seasonal shortages of the more appreciated foods (see Table S4).

### Species, families, growth forms, and ecology

From the more than 1500 species of vascular plants recorded for Guinea-Bissau [32], 436 have been reported in other West African countries for food purposes in two reference works (Useful Plants of West Tropical Africa [15], and Arbres, arbustes et lianes d’Afrique de l ‘Ouest [16]), and 94 of those species were previously reported in the country [29].

**Fig. 2:**
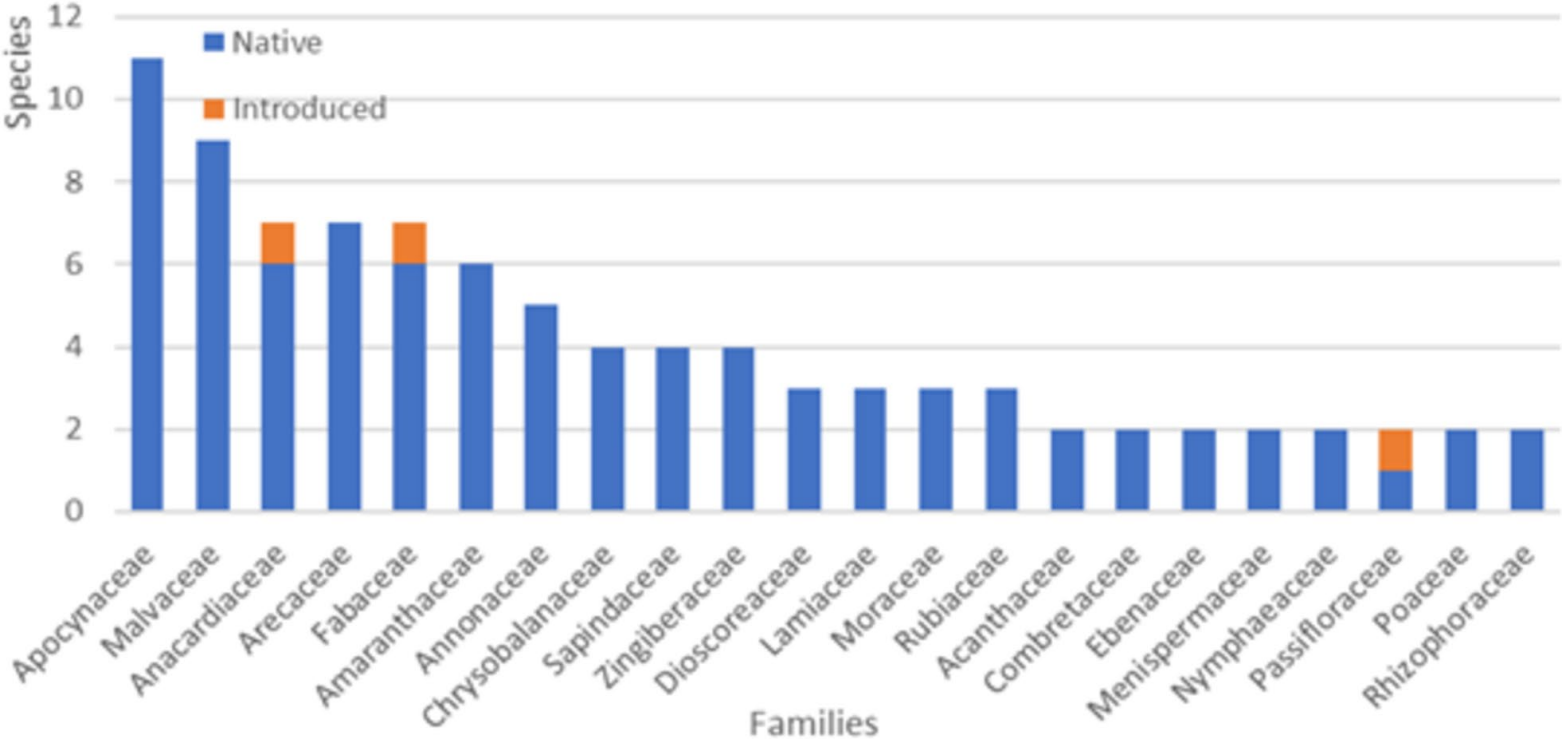
Families (with two or more species) of vascular plants represented in the wild edible flora of Guinea-Bissau

With our recent field work, it was possible to document the use of 21 further species, thus totaling 115 species of vascular plants used for food by local populations in Guinea-Bissau (Table S4; Fig. 2). Those species belong to 45 families and 89 genera; 111 are native species and four are introduced and naturalized (*Passiflora foetida* L.,* Physalis angulata* L.*, Spondias mombin* L.*, Trichosanthes cucumerina* L.). For about 60% (67) of the recorded WEPs, various medicinal uses had already been recorded in Guinea-Bissau [21, 26–28].

With 11 edible species recorded, Apocynaceae is the most represented family, with the majority of species growing as lianas. Seven of them are traded, and *S. senegalensis* and *Landolphia heudelotii* A.DC have a high socioeconomic importance (see Table 2), also being sold to neighboring countries. The Malvaceae, with nine edible species of trees, are an important family of WEPs, from which *A. digitata* and *Ceiba pentandra* (L.) Gaertn. presented the highest importance scores. Anacardiaceae, Arecaceae, and Fabaceae, each with seven species of WEPs in Guinea-Bissau, include important native species such as *Elaeis guineensis* Jacq. and *P. biglobosa*, or naturalized species such as *S. mombin* (Table 2).

**Fig. 3:**
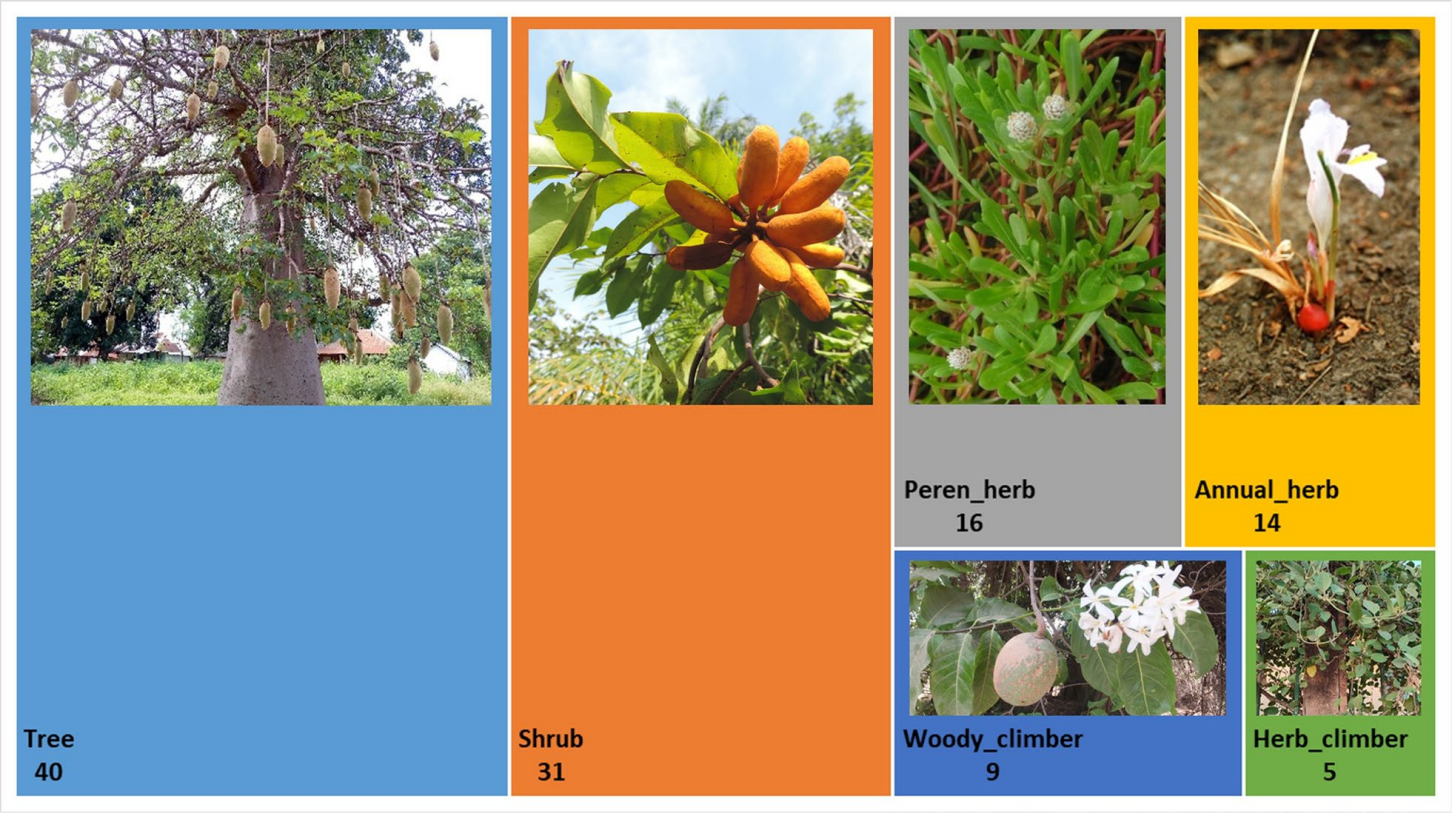
Treemap of growth forms and number of species of wild edible plants found in Guinea-Bissau, and images of examples: *Adansonia digitata* (Tree); *Uvaria chamae* (Shrub); *Gomphrena vermiculares* (Perennial herb); *Aframomum alboviolaceum* (Annual herb); *Saba senegalensis* (Woody climber); *Leptadenia lanceolata* (Herbaceous climber). (photos by LC and BI)

Concerning the growth forms, the woody plants dominate (Fig. 3). The tree and shrub habits corresponded to almost two-thirds of WEP species in Guinea-Bissau, and a considerable number of woody climbers were also recorded, mainly from the Apocynaceae family. The annual and perennial herbs accounted for about one quarter of the WEPs in the country, and five species of herbaceous climbers were recorded (Fig. 3).

Most of these plants grow in woodlands (e.g., *D. guineense, Parinari excelsa* Sabine*, S. mombin*) and in savannah woodlands (e.g., *Borassus aethiopum* Mart.*, P. biglobosa, Uvaria chamae* P.Beauv.) but many are also found in fallows (e.g., *Icacina oliviformis* (Poir.) J.Raynal, *Landolphia dulcis* (Sabine ex G.Don) Pichon,* Salacia senegalensis* (Lam.) DC.*, Sesamum radiatum* Thonn. ex Hornem.). Some species occur mainly around the villages, namely, *A. digitata, Borassus* spp., and *C. pentandra* (L.) Gaertn.

The data obtained in this study reveal a remarkable diversity of WEPs recorded in Guinea-Bissau, corroborating previous inventories conducted in the Tombali and Quinara regions [40]. This diversity is comparable to that reported in earlier studies carried out both in Guinea-Bissau and in neighboring West African countries, such as Senegal and the Guinea Republic, allowing the identification of both common usage patterns that enrich the regional understanding of food systems based on wild resources [e.g., 16, 52].

Species such as *A. digitata*, *D. guineense*, *P. biglobosa*, and *S. senegalensis* were consistently mentioned in earlier research [23, 30, 31], confirming their widespread importance in local diets and food culture. Beyond reaffirming these well-established species, this study contributes new insights by documenting a broader range of WEPs with nutritional and ecological relevance. Species like *Borassus akeassii* Bayton, Ouédr. & Guinko, *Lannea acida* A.Rich, *Hyphaene thebaica* (L.) Mart., *Mondia whitei* (Hook.f.) Skeels, *Psychotria peduncularis* (Salisb.) Steyerm., *Raphia sudanica* A.Chev., and *Strychnos spinosa* Lam. (see Table S4), although not entirely unknown, have received limited attention in previous investigations within the region. Their inclusion here reflects a more systematic approach to map underutilized biodiversity and traditional knowledge.

At the regional level, usage patterns are similar to those observed in rural areas of Senegal and the Guinea Republic, although variations occur in consumption frequency and preparation methods [52–55]. These differences may be linked to cultural, ecological, and accessibility factors. The comparison thus highlights both commonalities and local specificities that enrich the understanding of food dynamics in West Africa, in line with the floristic and ethnobotanical surveys compiled by Burkill [15] and Arbonnier [16].

### Plant parts and classes of use

**Fig. 4:**
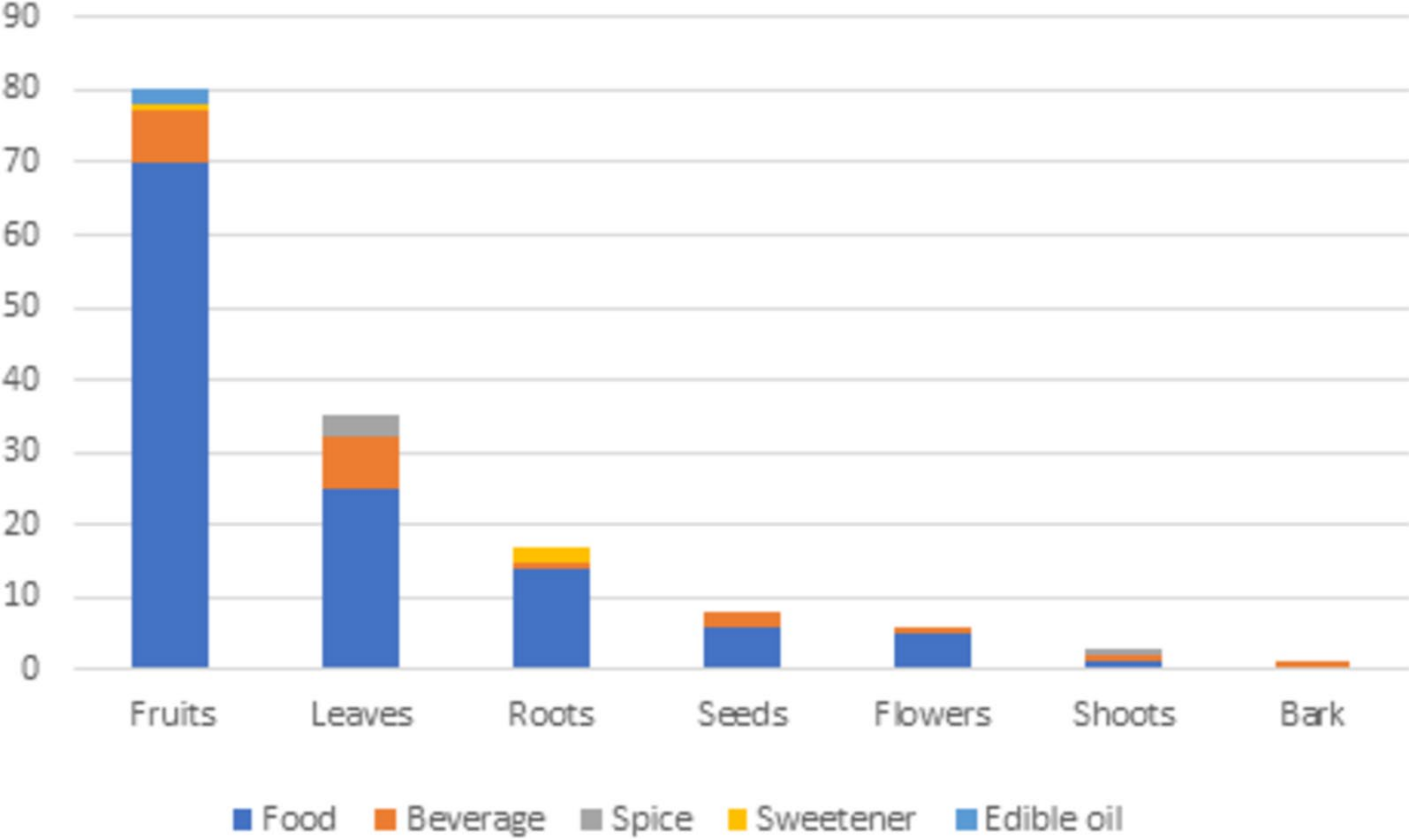
Number of species of wild edible plants of Guinea-Bissau according to their purposes and plant parts used

Analyzing the information on WEPs uses (Fig. 4), we highlight the number of those with edible fruits (70 species), most of them eaten fresh (*in natura*), and in some cases, also used to prepare beverages (7) or oil (2). The leaves of 30 species are consumed: 25 as food (e.g., *A. digitata*, *C. pentandra*, *Amaranthus* spp., *Hibiscus* spp.), seven in beverages (e.g., *Combretum micranthum* G.Don, *Piliostigma thonningii* (Schumach.) Milne-Redh.,* Terminalia macroptera* Guill. & Perr.), and three as condiments or spices (*Cymbopogon caesius* (Hook. & Arn.) Stapf*, Nelsonia canescens* (Thumb.) Spreng.*, Platostoma africanum* P.Beauv.) (Table 2). Flowers (5 species), seeds (6), and roots (14) are mainly used as cooked food. Different parts of the same plant are used from species such as *A. digitata* (leaf and fruit), *Xylopia aethiopica* (Dunal) A.Rich. (root and fruit), and *E. guineensis* (fruit and sap). These results are consistent with other studies carried out in Africa [e.g., 50, 52, 53, 54], which highlight the significant use of plants for food. However, neighboring countries report fewer WEPs than Guinea-Bissau: 87 species in the Republic of Guinea, without specification of uses [52], and in Senegal, a nationwide study [55] revealed that 27 species of wild leafy vegetables are consumed. From regional studies, we found 62 species consumed in the south-western region of Senegal [53] and 45 wild fruits consumed by the Malinke ethnic group in Senegal [54]. These surveys point to numbers lower than the number of WEPs found in this study, eventually due to the exhaustive nature of the study carried out throughout Guinea-Bissau.

### The socioeconomic importance of WEPs in Guinea-Bissau

**Fig. 5:**
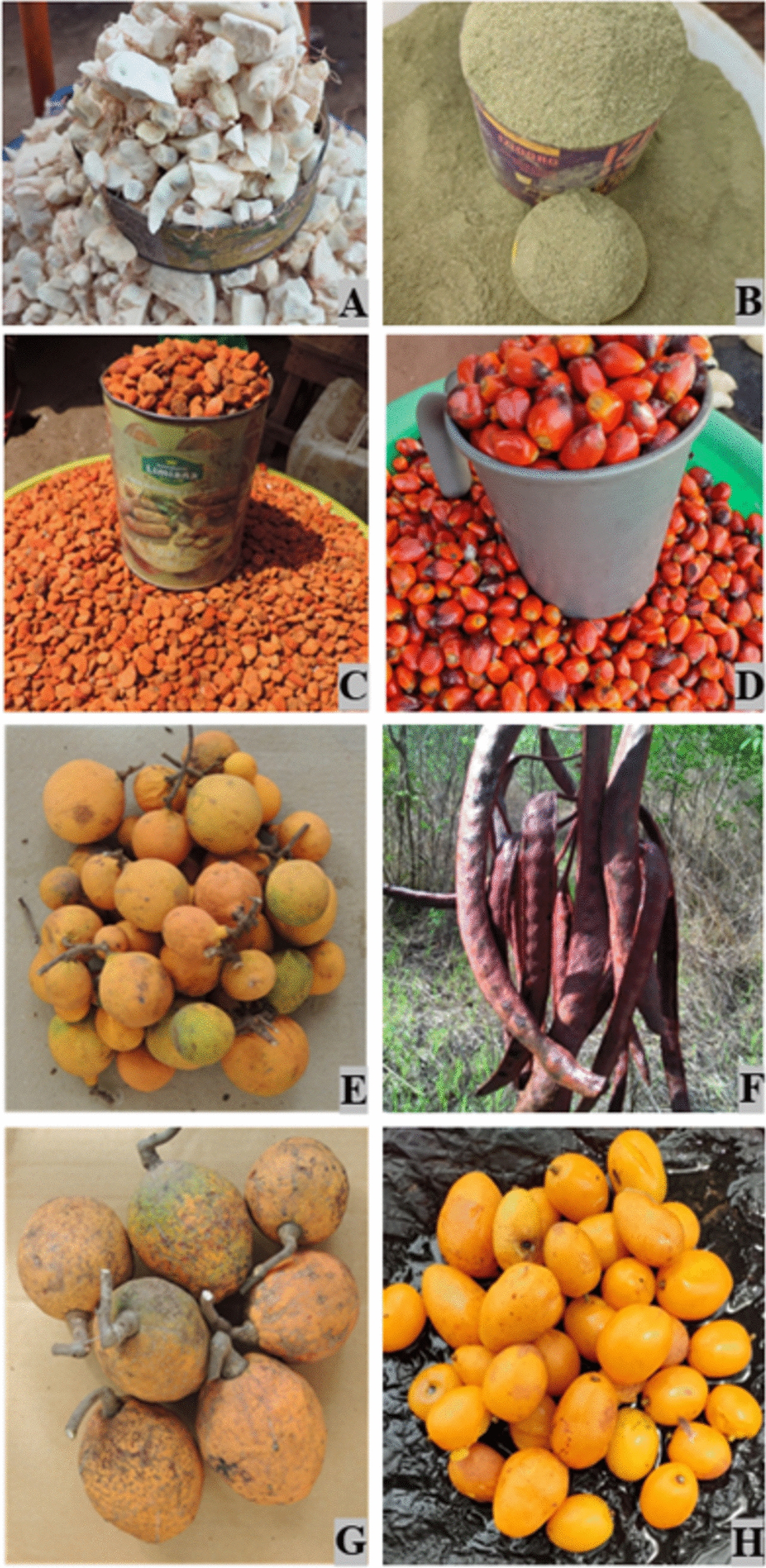
Used parts and derivatives of the eight most important wild edible species in Guinea-Bissau. **A** *Adansonia digitata*; **B** *Ceiba pentandra*; **C** *Dialium guineense*; **D** *Elaeis guineensis*; **E** *Landolphia heudelotii*; **F** *Parkia biglobosa*; **G** *Saba senegalensis*; **H** *Spondias mombin.* (photos by LC and BI)

Thirty-nine species of WEPs were recorded in local and city markets (Table 3). The sum of the scores of the six variables considered to assess the socioeconomic importance of the traded species (see Table 2) showed that eight species (≥ 20 points) are of very high socioeconomic importance: *A. digitata, C. pentandra, D. guineense, E. guineensis, L.a heudelotii, P. biglobosa, S. senegalensis,* and *S. mombin* (Table 3, Fig. 5). These species are traded across the country, some of them year-round, and contribute to the diets and to the livelihoods of populations. The edible part traded are the fruits from all of them except *C. pentandra*, from which the dried and ground leaves (locally known as *lalu*) are sold.

**Table 3:**
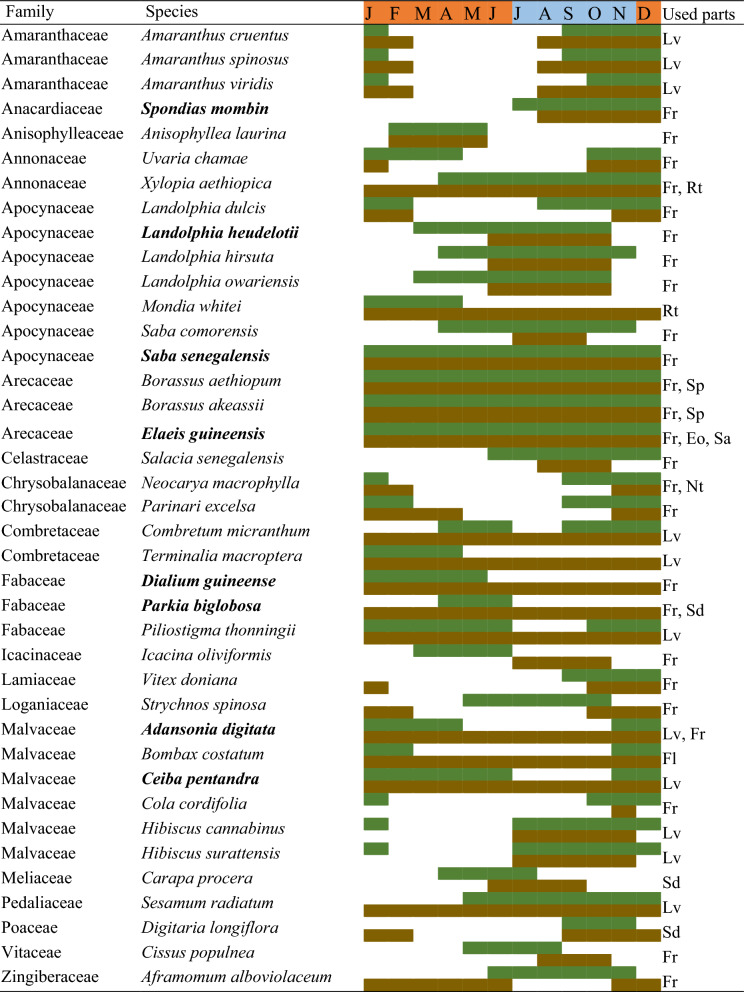
Periods of availability of the most important WEPs during the year—plant parts locally used (in green) and traded (in brown)

The WEP species in Guinea-Bissau with the highest socioeconomic value (see Table 2) are highlighted in bold

Abbreviations: *J* to *D*: months of the year. Used parts: *Eo* Edible oil, *Fl* Flowers, *Fr* Fruits, *Lv* Leaves, *Nt* Nut, *Rt* Roots and tubers, *Sa* Sap, *Sd* Seeds, *Sp* Sprout. Months of the dry season are shaded in orange, months of the wet season in blue. The WEP species in Guinea-Bissau with the highest socioeconomic value (see Table 2) are highlighted in bold

Another 17 species can be considered of high socioeconomic importance, scoring 15–19 points (Table 2). Most of the products traded for this group are the fruits, to be eaten fresh or to prepare beverages (e.g., *Anisophyllea laurina, X. aethiopica*), but leaves (*C. micranthum, P. thonningii* (Schumach.) Milne-Redh.*, S. radiatum, T. macroptera*) and roots (*M. whitei, X. aethiopica*) are also sold. The remaining 14 species are traded less frequently, and in some cases, only during limited periods of the year. These are mainly fruits, but also leaves, namely, from *Amaranthus* spp., cooked as vegetables (locally known as *djambô* or* bórbór*).

The socioeconomic importance of traded WEPs shows the role of the saleswomen (*bideiras*) and local markets (*lumus*) connecting rural and urban environments, and contributing to the preservation and appreciation of local knowledge, as well as its renewal. Moreover, these practices promote the economic sustainability of local communities, both rural and urban, and contribute to the conservation of WEPs [e.g., 30, 31].

### Patterns and trends in WEP consumption and trade

The consumption of WEPs is widespread in Guinea-Bissau and many of these plants are present in the daily lives of the populations across the various regions of the country. However, there are different usage patterns according to age (BI, personal observation). Although the elders have a larger knowledge about the uses and properties of plants, children consume a greater variety of edible plants, particularly fruits, than adults [20]. Often in groups, children in rural areas explore the surroundings of villages in search of edible plants and particularly fruits or sweet parts of plants, which Frazão-Moreira [19] refers to as children’s fruits (*fruto-di-minino*). The real contribution of these plants to children’s nourishment has not yet been studied. Still, they certainly play an important role in diversifying food sources and nutrients, not least because the variety of fruit or vegetables available for children is limited in many places. As some studies have shown, the importance of wild fruits consumed in Africa cannot be underestimated, particularly in terms of vitamins and minerals [56–58].

Some species, such as *Avicennia germinans* (L.) L., *Digitaria longiflora* (Retz.) Pers., *Dioscorea* spp., and *I. oliviformis* (see Table S4), are usually consumed only at times of the year when food is most scarce, serving as famine food, according to some authors [e.g., 59, 60]. These plants are generally not very tasty and often require lengthy processing before they can be consumed (e.g., repeated cooking), but they contribute to the food security of populations in periods of food scarcity. With the improvement of population food security and the commercialization of food products, the consumption of these resource foods appears to be decreasing, and many people report that these plants were consumed in the past but not anymore. However, there seems to be an important difference between plants that are consumed only in cases of extreme need and plants that are appreciated and valued but can be preserved to be consumed in times of greatest need, typically in the rainy season, especially in the months of August and September. Thus, the consumption of species such as *A. germinans*, *Dioscorea* spp. or *I. oliviformis* is considered a sign of poverty and lack of resources (Table S4). Conversely, it is common for families to collect and preserve bunches of *P. biglobosa* pods, as well as dried and crushed leaves of *A. digitata* or *C. pentandra*, to be consumed if necessary, which does not have the same connotation.

Introduced and naturalized species are currently used and many of them are also traded either locally or in the city markets. It is possible to find long-term naturalized species, such as *S. mombin*, and species recently introduced and undergoing a naturalization process, such as *Moringa oleifera* Lam. [61].

Several WEPs, namely, the eight species of greatest socioeconomic value mentioned above, are in great demand in urban markets in Guinea-Bissau, and are also exported to neighboring countries, especially Senegal and the Gambia [30, 38, 44]. On the other hand, some plant products are imported from the Republic of Guinea due to the high demand from migrants from this country living in Guinea-Bissau, such as dried leaves pounded into powder (*lalu*) of *A. digitata, Bombax costatum* Pellegr. & Vuillet, *C. pentandra*, and the almonds of *Neocarya macrophylla* (Sabine) Prance ex F.White. Some of these products vary greatly in price throughout the year and can reach high values during periods of greatest scarcity. For example, a single fruit of *S. senegalensis* (locally known in Guinea-Bissau Creole as *fole-lifanti*, Fig. 5G) can cost up to 500 or even 1000 XOF (USD 0.88–1.75) at the Bandim market in Bissau. Bearing in mind that the common monthly salary in Guinea-Bissau is roughly 50.000–250.000 XOF, and that the price of the primary food, rice, is 300–600 XOF/Kg (USD 0.6–1.05), it becomes clear how much WEPs are valued in urban locations.

The trade of WEPs in city markets is usually carried out by intermediaries, who go to the villages at harvest time to buy the products at relatively low prices. Transport to urban centers is costly and often difficult due to deficient infrastructure, for example, bad road conditions, especially in the rainy season. Furthermore, the transport of wild food products to urban markets is taxed by the forestry authorities [31]. However, this strong demand for WEPs in urban markets and neighboring countries promotes the collection of these plants in rural areas, mainly by young people and women, and allows for some family economic relief.

The trade of WEPs in West African markets highlights the central role of fruits and leaves in local commerce. In Burkina Faso [62] and Mali [63, 64], for example, the commercialization of species such as *A. digitata* and *D. guineense* has been documented in both urban and rural markets, as also revealed by our study. Furthermore, our study, together with research conducted in Senegal [53], emphasizes the importance of vine fruits such as *Saba* spp. and *L. heudelotii* in local markets. In the forest zone of the Republic of Guinea [65], *P. biglobosa* is traded in local markets, alongside other species native to tropical Africa that were not recorded in our survey, including *Piper guineense* Schumach. & Thonn., *Garcinia kola* Heckel, *Ricinodendron heudelotii* (Baill.) Heckel, and *Beilschmiedia mannii* (Meisn.) Benth. & Hook.f. ex B.D.Jacks. In summary, in local and urban markets in Guinea-Bissau, WEPs are common products and appear to be well accepted by consumers. However, for several reasons, only a part of the WEPs locally consumed in rural areas are harvested and traded in city markets. Many plants and plant parts are hard to preserve and cannot be transported to the markets, others have no demand, or the price offered is not attractive for collectors or sellers.

### WEPs seasonality and food security

The comprehensive results presented in Table 3 show the availability of WEPs along the year, plant parts used, and periods of trade in Guinea-Bissau. Some WEPs are available year-round—e.g., *B. aethiopum, B. akeassii, E. guineensis, S. senegalensis*—the latter being two of the most important ones in the country. In contrast, other fruits are clearly seasonal and harvested during the rainy season (e.g., *Carapa procera* DC*, S. mombin*), or in dry months (e.g., *B. costatum, D. guineense, P. biglobosa*), or for a few months in both seasons (e.g., *L. heudelotii*).

There is a pattern of availability related to plant families. For example, *E. guineensis* and the *Borassus* species (Arecaceae) are available for consumption year-round, and their products are easily preserved, while the edible parts of Amaranthaceae (mainly the fresh leaves) and the Chrysobalanaceae (fruits) are quite seasonal, being absent at the end of the dry season and the beginning of the rainy season (see Table 3).

The leafy vegetables are also seasonal: for instance, *Amaranthus* spp. can be harvested for 4–5 months during the dry season, and *Hibiscus* spp. for 6–7 months, especially during the rainy season. Their nutritional properties have already been evaluated [23, 66], showing their dietary importance, particularly in terms of minerals and antioxidants.

Many WEPs, especially fruits, have seasonal availability but can be found on the market at any time of year, such as *A. digitata, C. micranthum, D. guineense, E. guineensis,* and *X. aethiopica*. This supply is possible because their products are generally dry and can be easily kept at room temperature, allowing them to be stored for long periods in the urban centers where the markets are located [67]. It can therefore be concluded that, as in other similar African contexts [53, 54], many WEPs are available year-round, and are therefore important and reliable nutritional resources.

Our field and market surveys enabled us to identify several challenges to guarantee food security in Guinea-Bissau, namely, during the rainy season months (mainly August and September), when the greatest food scarcity affects rural populations, since the food produced in the previous production cycle has already been consumed and new production is not yet available. The WEPs available throughout the year (see Table 3) can represent an important strategy for the rural population to mitigate food insecurity and malnutrition in Guinea-Bissau. These species represent an accessible and nutritious source of food, especially during periods of scarcity or in communities with limited access to conventional agricultural products. Their integration into local food systems strengthens food sovereignty and reduces dependence on imports [30, 31].

### Nutraceuticals and functional foods

Our results indicate that many of the plants used as food also have properties and uses to prevent and treat diseases and health conditions. For instance, the West African Herbal Pharmacopoeia [68] acknowledges the medicinal value of several species identified in this study as edible, namely, *Annona senegalensis* Pers., *P. thonningii* (Schumach.) Milne-Redh., *T. macroptera*, *Vitex doniana* Sweet, and *Ximenia americana* L. and, from the 39 marketed WEPs, 34 are used for medicinal purposes in the country (Table 2), although sometimes the parts of the plant used for the two purposes are different. Moreover, many of these WEPs can be considered as nutraceutical plants or functional foods [e.g., 69, 70], i.e., food plants whose consumption is beneficial for health.

In most cases, local people do not consume WEPs for medicinal purposes or because they have pharmacological or disease-preventing effects. However, different studies demonstrate the medicinal relevance of these species, as shown by the properties of some of the most important WEPs we found. Several parts of the baobab (*A. digitata*) reveal antioxidant, antiviral, and anti-inflammatory properties [71, 72]. The shell and, especially, the pulp and seed of velvet tamarind, *D. guineense*, are good sources of nutrients and could serve as natural antioxidants if incorporated in human diet [73]. Also, Gernah et al. [74] demonstrated that the fruit pulp of the African locust bean (*P. biglobosa*) is a good source of macro- and micronutrients, and can favorably compete with most cereals and legumes; moreover, it is a medicinal species whose importance is well recognized both regionally and internationally (see Table 2).

The nutritional value of the fruit pulp of *S. senegalensis* can positively contribute to the dietary balance of populations in Burkina Faso and serves as a remedy for specific nutritional deficiencies in individuals with disabilities [75]. The fruits of *S. mombin* contain significant proportions of phenolic compounds, vitamins, and minerals; further research is necessary to help improving its value chain and promote its consumption [76].

Although local people can use WEPs regularly for beverages without actually considering them medicines (*mesinho*), they perceive their importance for health. This seems to be the case of the drink prepared with the fruits of *X. aethiopica* [20, 67], which contain substances with expectorant, antispasmodic, and cough-sedative properties, as well as antimicrobial properties [77, 78].

In addition to the beneficial effect in terms of food diversity and nutritional richness, there are no known negative effects of consuming WEPs. Based on their accurate traditional ecological knowledge, the rural population of Guinea-Bissau avoids consuming toxic plants and carries out the necessary processing before consuming plants identified as WEPs.

Many neglected and underutilized species are often highly nutritious, and enhance human health and well-being. When these species are included in a varied diet, they help to offset malnutrition, hidden hunger, overweight and obesity. In short, the great importance of WEPs as nutraceuticals and functional foods should not be overlooked.

### Conservation of WEP

During the field surveys, it was noticed that some trees, palms, and lianas (e.g., of *A. digitata, A. laurina, Borassus* spp., *D. guineense, E. guineensis, P. biglobosa, S. senegalensis,* or *X. aethiopica*) are beginning to be cared for, and that the populations are increasingly aware of the need to protect the forests where they occur, for example, through the establishment of community forests [31, 79]. This trend, although recent and not yet widespread, can contribute to the conservation and sustainable use of natural resources in Guinea-Bissau. For instance, *P. biglobosa*, whose socioeconomic importance is presently recognized, is being actively planted by women in northern regions of the country because of the value of its fruits (until recently, the trees were cut down and used for charcoal production). Another recent practice is the growing of *S. senegalensis* in homegardens in Bissau, both as ornamental plant and for its edible fruits, and the same is happening for *A. laurina* and *S. mombin*. Also, we found that *A. digitata* and *C. pentandra*, although not actually cultivated, are cared for and preserved in the villages since they are very young plants, due to their sociocultural importance.

Apparently, the socioeconomic valorization of the WEP species has been significantly contributing to raise awareness about the importance of their conservation. However, studies are lacking to confirm whether harvesting in forests and savannas has increased for local consumption, and whether the increasing trade will impact their sustainability [31].

## Conclusions

The present study documented 115 WEPs in Guinea-Bissau, including 21 species recorded for the first time as WEPs in the country. Over one-third of these plants are traded in local, city, and international markets. Conversely, only very few introduced and naturalized WEPs are consumed or sold in markets. The findings of this study have relevant implications for environmental issues, food security, and public health in Guinea-Bissau. This expansion of the knowledge on WEPs for Guinea-Bissau represents a significant advancement, as it reveals undocumented local knowledge and reinforces the need for ongoing ethnobotanical and phytochemical studies. Furthermore, a greater appreciation of these species was observed in communities with limited access to markets, suggesting a direct relationship between economic vulnerability and reliance on wild resources.

From an environmental perspective, the sustainable use of WEPs contributes to biodiversity conservation and the maintenance of ecosystem services, such as soil regeneration, pollination, and agroecosystem resilience in the face of climate change [40, 47].

WEPs play a dual socioeconomic role: as essential food sources for rural communities and as a valuable source of income along the entire value chain. Their market prices differ significantly between local and city markets, and even more in foreign countries, with some species reaching high prices during periods of scarcity. This has led many rural collectors, primarily women and young men, to recognize the economic potential of WEPs. The growing demand, along with commercial viability, may contribute to the conservation of high-value species, such as *A. digitata, D. guineense, E. guineensis, L. heudelotii, P. biglobosa, S. senegalensis,* and *S. mombin*.

Some species, like *S. senegalensis*, appear to be undergoing a domestication process, with some plants being cultivated in local backyards, also showing potential for inclusion in agroforestry systems. Furthermore, community forests are increasingly seen as sources of marketable non-timber forest products, with the harvest and trade of these plants offering opportunities for empowering rural communities, especially women and young men.

The availability and preservation of many WEPs throughout the year make them important for dietary diversification and food security. Many of the 39 species that are commercialized are available year-round and can be preserved for long periods, which is relevant for both food security and family income. Some plants are universally appreciated, and their use is rooted in Guinean culture. They are generally products that are easy to preserve, available year-round and often used as ingredients in local cuisine. Other plants are occasionally consumed in rural areas when they are available, but they are generally rarer, more difficult to preserve and are not commercialized.

Interestingly, children in rural areas tend to eat a wider variety of plants than adults, especially fruits. Although the impact of this consumption on children’s diet is not yet fully understood, it likely plays an important role in diversifying food sources. More in-depth studies are also needed to explore the consumption patterns of WEPs, particularly those related to age, gender, and cultural and ethnolinguistic drivers.

Some WEPs are consumed mainly during the lean months when food availability is low. These “famine plants” are not particularly appetizing but serve an important function in times of food scarcity. As socioeconomic conditions improve, the reliance and knowledge on these plants tends to disappear. However, the valorization of WEPs should not be seen merely as a survival strategy, but as an opportunity to promote more sustainable, inclusive, and locally rooted development models. Their integration into food education programs, community health initiatives, and environmental management activities can strengthen food sovereignty and contribute to local resilience in the face of ecological and economic crises.

This study demonstrates the socioeconomic importance of WEPs in Guinea-Bissau and their potential to enhance food security and dietary diversity in rural areas. The sustainable use of these species can improve local livelihoods while supporting the conservation of biodiversity and ecosystem services.

Although considerable efforts were made to document and synthesize information on WEPs, some limitations remain. Reported nutritional properties are based largely on local knowledge and require laboratory validation. Further research is also needed on their market potential and the ecological impacts of harvesting. Despite these limitations, this work provides a valuable foundation for deepening the understanding of WEPs and their role in the livelihoods and cultural heritage of Guinea-Bissau’s rural communities.

## Supplementary Information

Supplementary file1

## Data Availability

Data are provided within the manuscript.

## Abbreviations

INE: Instituto Nacional de Estatítica (National Institute of Statistics of Guinea-Bissau)
INEP: Instituto Nacional de Estudos e Pesquisa (National Institute for Studies and Research, Guinea-Bissau).
LISC: Code of the Herbarium of the University of Lisbon, Portugal
POWO: Plants of the World Online
PROTA: Plant Resources of Tropical Africa website
WEP: Wild edible plant
WEPs: Wild edible plants
XOF: West African CFA franc
