# Supplementary material for: Diversity of wild edible plants of Guinea-Bissau (West Africa): traditional uses and trade
Source: J Ethnobiol Ethnomed. 2025 Dec 24;21:88. doi: 10.1186/s13002-025-00825-w (PMC12729238; doi:10.1186/s13002-025-00825-w)
Supplement: Supplementary file 1 — Supplementary file1 [file 13002_2025_825_MOESM1_ESM.docx]

**Supplementary data**

**Diversity of wild edible plants of Guinea-Bissau (West Africa): traditional uses and trade**

Bucar Indjai^1,2,3,4^, Amélia Frazão-Moreira^5^, Pedro Segurado^1,6^, Maria Manuel Romeiras^2,4,6^*, Luís Catarino^4^*

^1^ CEF - Forest Research Centre, School of Agriculture, University of Lisbon, Tapada da Ajuda, 1349-017 Lisboa, Portugal.

^2^ LEAF - Linking Landscape, Environment, Agriculture and Food, School of Agriculture, University of Lisbon, Tapada da Ajuda, 1349-017 Lisboa, Portugal.

^3^ INEP - Instituto Nacional de Estudos e Pesquisa (INEP), Avenida dos Combatentes da Liberdade da Pátria, Complexo Escolar 14 de Novembro - Bissau, Guiné-Bissau - Caixa Postal 112.

^4^ CE3C - Centre for Ecology, Evolution and Environmental Changes & CHANGE—Global Change and Sustainability Institute, Faculty of Sciences, University of Lisbon, Campo Grande 1749-016 Lisboa, Portugal

^5^ Centre for Research in Anthropology (CRIA) / School of Social Sciences and Humanities, NOVA University of Lisbon (NOVA FCSH) and IN2PAST Associate Laboratory, Av. de Berna, 26 C, 1069-061, Lisboa, Portugal

^6^ TERRA Associate Laboratory, School of Agriculture, University of Lisbon, Tapada da Ajuda, 1349-017 Lisboa, Portugal.

* Corresponding authors: mmromeiras@isa.ulisboa.pt; lmcatarino@fc.ul.pt

**Supplementary Table S1.** Coordinates and general characteristics of the locations where markets were surveyed in 2022-2024 (see also the map in Figure 1). The only city market surveyed is Bandim, in Bissau; all the others are rural markets ("*lumus*").

| **Markets** | **Lat N** | **Long W** | **Region** | **Market type*** | | **Date visited** |
| --- | --- | --- | --- | --- | --- | --- |
| **City daily market** | |  |  |  |  | |
| Bandim, Bissau | 11°51'29’’ | 15°35'56’’ | Bissau capital | Main daily market | | January 2022 to March 2024 |
| **Local weekly markets** | |  |  |  |  | |
| Bantandjan | 12°02'54’’ | 14°50'34’’ | East | Local market | | July, 2024 |
| Bigene | 12°26'23’’ | 15°32'18’’ | North | Local market | | May, 2024 |
| Bissorã | 12°13'24’’ | 15°27'03’’ | North | Wholesale market | | April, 2024 |
| Buba | 11°35'46’’ | 14°58'46’’ | South | Local market | | May, 2024 |
| Bubaque | 11°17'59’’ | 15°49'36’’ | Bijagós Islands | Local market | | April, 2024 |
| Bula | 12°06'26’’ | 15°43'05’’ | North | Wholesale market | | May, 2024 |
| Calequisse | 12°04'02’’ | 16°13'29’’ | North | Local market | | July, 2024 |
| Cambadju | 12°40'24’’ | 14°45'29’’ | East | Frontier market | | July, 2024 |
| Cameconde | 11°04'46’’ | 14°59'37’’ | South | Frontier market | | June, 2024 |
| Canchungo | 12°03'59’’ | 16°01'54’’ | North | Wholesale market | | January, 2022 |
| Cubumba | 11°22'16’’ | 15°09'25’’ | South | Local market | | May, 2024 |
| Cuntabane | 11°33'01’’ | 14°41'14’’ | South | Frontier market | | May, 2024 |
| Djolmete | 12°13'11’’ | 15°51'30’’ | North | Wholesale market | | January, 2022 |
| Elia | 12°19'36’’ | 16°24'00’’ | North | Frontier market | | January, 2022 |
| Enxudé | 11°50'09’’ | 15°25'31’’ | South | Local market | | May, 2024 |
| Gãmamudo | 12°01'59’’ | 15°05'23’’ | North | Local market | | July, 2024 |
| Gandembel | 11°22'29’’ | 14°47'48’’ | South | Wholesale market | | June, 2024 |
| Guiledje | 11°18'32’’ | 14°50'53’’ | South | Wholesale market | | June, 2024 |
| Ilha de Komo | 11°11'52’’ | 15°20'03’’ | South Islands | Local market | | May, 2024 |
| Ingoré | 12°23'46’’ | 15°47'28’’ | North | Frontier market | | May, 2024 |
| Jacaradje | 11°48'22’’ | 14°49'27’’ | East | Wholesale market | | May, 2024 |
| Mafanco | 12°15'53’’ | 14°26'21’’ | East | Local market | | July, 2024 |
| Mansaba | 12°17'41’’ | 15°10'13’’ | North | Wholesale market | | May, 2023 |
| Mansoa | 12°04'04’’ | 15°19'03’’ | North | Wholesale market | | August, 2024 |
| Nhacra | 11°57'33’’ | 15°32'17’’ | North | Wholesale market | | January, 2023 |
| Pitche | 12°19'36’’ | 13°57'14’’ | East | Frontier market | | July, 2024 |
| Quebo | 11°32'13’’ | 14°46'00’’ | South | Frontier market | | June, 2024 |
| São Domingos | 12°25'09’’ | 16°12'14’’ | North | Frontier market | | January, 2022 |
| Suzana | 12°18'22’’ | 16°28'28’’ | North | Frontier market | | January, 2022 |
| Tantan-Cossé | 12°15'08’’ | 14°32'29’’ | East | Local market | | July, 2024 |
| Tchalana | 12°01'15’’ | 15°12'43’’ | North | Wholesale market | | August, 2024 |

*Market types: Frontier market - markets in border areas, where products are traded for sale in neighboring countries, namely Senegal and the Republic of Guinea; Local market - market where products are sold primarily for local consumption; Wholesale market - market where products are sold in large quantities frequently to be resold in the cities.

**Supplementary Table S2.** Market surveys. Field questionnaire on the occurrence and trade of wild edible plants in Bissau’s city market and in rural markets (“*lumus*”) of Guinea-Bissau (market details provided in Table S1).

**Field Questionnaire:**

**Date:**  _____________ **Market name:**  _____________________ **Geographical location (N/W):** ______________________

***Interviewee Approach***

- Brief presentation of the study’s objectives
- Request for availability to participate
- Verbal informed consent for participation and data use

***Interviewee Information***

- Name: ________________________________________________________________________________________
- Age: __________________________________________________________________________________________
- Gender: _______________________________________________________________________________________
- Occupation: ____________________________________________________________________________________
- Ethnicity: ______________________________________________________________________________________

***Activity in the Market (Lumo)***

- ( ) Collector
- ( ) Seller and collector
- ( ) Consumer

***Observation and Identification of Traded Products (by researcher)***

- Local name of the product (species): ________________________________________________________________
- Parts used: ____________________________________________________________________________________
- Preparation method: _____________________________________________________________________________
- Harvest season: _________________________________________________________________________________
- Harvest location: ________________________________________________________________________________
- Availability: ____________________________________________________________________________________
- Preservation method: _____________________________________________________________________________
- Product price (FCFA): ____________________________________________________________________________

Thank you for your cooperation.

**Interviewer:** Bucar Indjai

**National Institute for Studies and Research (INEP), Guinea-Bissau**

**Center for Environmental Studies and Appropriate Technology (CEATA), Guinea-Bissau**

**Supplementary Table S3.** Comprehensive data on botanical vouchers of wild edible plants from Guinea-Bissau, housed at the LISC Herbarium (University of Lisbon). The table includes the following information: Voucher Code, Family, Species, Collectors, Collector Number, Collection Date, Country, and Collection Location in Guinea-Bissau.

| **Voucher code** | **Family** | **Species** | **Collectors** | **Number** | **Date** | **Location** |
| --- | --- | --- | --- | --- | --- | --- |
| LISC131167 | Acanthaceae | *Avicennia germinans* (L.) L. | L. Catarino; M. A. Djaló | 325 | 11/12/1997 | Região de Quinara. Parque Natural das Lagoas de Cufada. Rio Uassadu. 11º 44' 45''N / 15º 09' 09''W |
| LISC131168 | Acanthaceae | *Nelsonia canescens* (Thumb.) Spreng. | L. Catarino & Q. Bancessi | 1108 | 17/05/2000 | Região de Cacheu, Setor de São Domingos, pero de Suzana 12º 18' 44"N / 16º 29' 20"W |
| LISC131169 | Aizoaceae | *Sesuvium portulacastrum* (L.) L. | A. Moreira | 342 | 14/04/1996 | Catanhéz, Catchamba Nalu |
| LISC131170 | Amaranthaceae | *Alternanthera nodiflora* R.Br. | M.F.P. Basto; U.Candé; Q. Bancessi & Q. Correia | 124 | 08/06/1989 | Provincia Leste, Região de Gabu, tabanca Sonaco. |
| LISC131171 | Amaranthaceae | *Amaranthus cruentus* L. | J. Alves Pereira | 3200 | 13/09/1962 | Região de Gabu, Piche |
| LISC131172 | Amaranthaceae | *Amaranthus spinosus* L. | M. Adelia Diniz, C.Duarte & Umaro Candé | 353 | 19/09/1990 | Provincia Leste, Contuboel, tabanca Fajanquito |
| LISC131173 | Amaranthaceae | *Amaranthus viridis*L. | J.A. Guerra | 3983 | 22/01/1963 | Fá, região de Bafatá |
| LISC131174 | Amaranthaceae | *Celosia argentea*L. | A. Moreira | 300 | 26/11/1994 | Catanhez, Catomboi |
| LISC131175 | Amaranthaceae | *Gomphrena vermicularis*L. | M. Adelia Diniz & A. E. Gonçalves | 1854 | 16/04/1997 | Região de Cacheu, São Domingos, Varela. |
| LISC128656 | Anacardiaceae | *Lannea acida*A.Rich | A. Moreira | 43 | 09/12/1993 | Iemberém, Caminho de Cadique M'bitna e Cadique Nalu |
| LISC003047 | Anacardiaceae | *Lannea nigritana* (Scott Elliot) Keay | Catarino & Bancessi | 391 | 02/04/2000 | Região de Gabu, setor de Boé, Picada Contabane - Boé, junto a Dandu. |
| LISC128702 | Anacardiaceae | *Lannea velutina*A.Rich. | E.S. Martins & L. Catarino | 1201 | 03/06/1996 | Parque Nacional das Lagoas de Cufada, 2 km de Fulacunda para Uana Porto. |
| LISC128773 | Anacardiaceae | *Pseudospondias microcarpa* (A.Rich.) Engl. | M. Campos, B. Indjai, M. Andre | 121 | 31/10/1996 | Ambanha, Ilha de Bubaque |
| LISC128791 | Anacardiaceae | *Sclerocarya birrea*(A.Rich.) Hochst. | M.L. Gonçalves, M.F.P. Basto, U.Candé, Q. Bancessi & F. Sané | 83 | 02/11/1988 | Provincia de Leste, Região de Gabú, Setor de Pirada, Cam Djufa. |
| LISC003054 | Anacardiaceae | *Sorindeia juglandifolia* (A.Rich.) Planch. ex Oliv. | L. Catarino & Q. Bancessi | 482 | 05/04/2000 | Região de Gabu, Setor de Boé, perto de Lugadjole. 11º 48' 42"N / 13º 51' 44"W |
| LISC003056 | Anacardiaceae | *Spondias mombin* L. | L. Catarino & Q. Bancessi | 701 | 15/04/2000 | Região de Cacheu, setor de Cacheu. 12º 15' 47"N / 16º 01' 37"W |
| LISC003008 | Anisophylleaceae | *Anisophyllea laurina* R.Br. ex Sabine | L. Catarino & Q. Bancessi | 779 | 26/04/2000 | Região de Bolama, setor de Caravela. 11º 27' 52"N / 15º 55' 49"W |
| LISC092489 | Annonaceae | *Annona glabra*L*.* | Amélia Moreira | 38 | 09/12/1993 | Iemberém, Cadique, junto ao hospital novo. |
| LISC092497 | Annonaceae | *Annona glauca*Schumach. & Thonn. | A. Moreira | 260 | 02/07/1994 | Cantanhéz, Canamine, Lala |
| LISC093010 | Annonaceae | *Annona senegalensis*Pers. | M. Adélia Diniz, A.E. Gonçalves & L. Catarino | 991 | 17/10/1995 | Região de Cacheu, setor de São Domingos, seção de Varela, Bairro Lala. 12º 17' 23" N / 16º 34' 25"W |
| LISC093178 | Annonaceae | *Uvaria chamae*P.Beauv. | Adelia Diniz & A.E. Gonçalves | 1908 | 18/04/1997 | Região de Cacheu, setor de São Domingos, Varela, Catão. 12º 17' 22" N / 16º 32 17"W |
| LISC093260 | Annonaceae | *Xylopia aethiopica* (Dunal) A.Rich. | L. Catarino & Q. Bancessi | 879 | 02/05/2025 | Região de Bolama, setor de Uno, Ilha de Orango. 11º 10' 26"N / 16º 08' 34"W |
| LISC131187 | Apocynaceae | *Landolphia dulcis* (Sabine ex G.Don) Pichon | M. Adélia Diniz & A. E. Gonçalves & L. Catarino | 1227 | 19/10/1995 | Região de Cacheu, setor de São Domingos, tabanca Candemba. 12º 19' 7,8"N / 16º 27' 26,3"W |
| LISC131182 | Apocynaceae | *Landolphia heudelotii* A.DC. | M. Adélia Diniz & A.E. Gonçalves | 1812 | 15/04/1997 | Região de Cacheu, Setor de São Domingos, Varela. 12º 19' 22"N / 16º 23' 57"W |
| LISC131180 | Apocynaceae | *Landolphia hirsute* (Hua) Pichon | M. Adélia Diniz, I. Moreira & A. Moreira | 967 | 16/11/1994 | Porto de Cananime, margem esquerda do rio Cacine |
| LISC131184 | Apocynaceae | *Landolphia incerta* (K.Schum.) J.G.M.Pers. | J. Alves Pereira | 2827 | 16/01/1962 | Bedanda, Cantanhez |
| LISC131183 | Apocynaceae | *Landolphia owariensis* P.Beauv. | Fodé Sané | 38 | 22/03/1984 | Carabana, Região de Cacheu |
| LISC131185 | Apocynaceae | *Leptadenia lanceolata* (Poir.) Goyder | M. Adélia Diniz & A.E. Gonçalves | 1823 | 16/04/1997 | Região de Cacheu, Setor de São Domingos, Varela. 12º19'22"N / 16º 34' 11" W |
| LISC131181 | Apocynaceae | *Mondia whitei*(Hook.f.) Skeels | J. Alves Pereira | 3114 | 10/08/1962 | Cuntubó |
| LISC131179 | Apocynaceae | *Saba comorensis* (Bojer ex A.DC.) Pichon | J.A. Guerra | 4003 | 04/o4/1963 | Fá, região de Bafatá |
| LISC131178 | Apocynaceae | *Saba senegalensis* (A.DC.) Pichon | E.S. Martins & L. Catarino | 1674 | 02/05/2001 | Quinara, Parque Nacional das Lagoas de Cufada. 11º38'16"N / 15º06'51"W |
| LISC131176 | Apocynaceae | *Tacazzea apiculata*Oliv. | E.S. Martins & L. Catarino | 1232 | 04/06/1996 | Parque Natural das Lagoas de Cufada. 11º 41' 40,2"N / 15º 09' 27,4"W |
| LISC131177 | Apocynaceae | *Voacanga africana*Stapf | M. Campos, B. Indjai, M. André | 128 | 31/10/1996 | Ambanha, Ilha de Bubaque |
| LISC131186 | Araliaceae | *Cussonia arborea*Hochst. ex A.Rich. | J. Alves Pereira | 2290 | 09/12/1961 | Piche-Burumtuma |
| LISC131188 | Arecaceae | *Borassus aethiopum*Mart. | J. Alves Pereira | 1058 | 28/12/1960 | Mansaba, Cucutó |
| LISC131236 | Arecaceae | *Borassus akeassii* Bayton, Ouédr. & Guinko | Bucar Indjai | 228 | 03/05/2024 | Região de Cacheu, Setor de Canchungo, junto a povoação. 12. 07454 N / 16. 04477W |
| LISC061874 | Arecaceae | *Elaeis guineensis*Jacq. | M. Adélia Diniz, A.E. Gonçalves & L. Catarino | 1031 | 17/10/1994 | Região de Cacheu, Setor de São Domingos, seção de Varela. 11º17' 56,8"N / 16º 33'44,4"W |
| LISC131189 | Arecaceae | *Hyphaene thebaica*(L.) Mart. | J. Espirito Santo | 2425 | 28/01/1988 | Estrada entre Mansoa e Mansaba |
| LISC061879 | Arecaceae | *Phoenix reclinata*Jacq. | E.S. Martins & L. Catarino | 1404 | 23/11/1997 | Parque Natural das Lagoas de Cufada. 11º 44' 23" N / 14º 57' 30"W |
| LISC131190 | Arecaceae | *Raphia palma-pinus* (Gaertn.) Hutch. | F. Raimundo e J.A.Guerra | 549 | 15/12/1960 | Teixeira Pinto, Cacheu, Pijame |
| LISC131191 | Arecaceae | *Raphia sudanica*A.Chev. | Bucar Indjai | 226 | 08/01/2023 | Região de Gabú, Setor de Pitche, junto a estrada de Pitche para Cambore. Bolanha de Bidigor, cerca de 2 km para Pitche. 12º 21' 52.336699 N / 13º 56' 59.542800W |
| LISC120870 | Celastraceae | *Salacia senegalensis*(Lam.) DC. | E.S. Martins & L. Catarino | 1211 | 04/06/1996 | Parque Natural das Lagoas de Cufada. Buba-Fulacunda. 11º 42' 28"N / 15º 04' 45"W |
| LISC003007 | Chrysobalanaceae | *Chrysobalanus icaco*L. | L. Catarino & Q. Bancessi | 850 | 01/05/2000 | Região de Bolama, setor de Uno; Ilha de Orango. 11º 10' 26"N / 16º 08' 36"W |
| LISC131192 | Chrysobalanaceae | *Neocarya macrophylla* (Sabine) Prance ex F.White | M. Adélia Diniz & A. E. Gonçalves | 1778 | 15/04/1997 | Região de Cacheu, setor de São Domingos, desvio para Elia. 12º 19' 22"N / 16º 23' 57"W |
| LISC131193 | Chrysobalanaceae | *Parinari curatellifolia* Planch. ex Benth. | J. Alves Pereira | 1227 | 07/01/1961 | Olossato, regio de Oio |
| LISC131194 | Chrysobalanaceae | *Parinari excelsa* Sabine | Amélia Moreira | 229 | 01/06/1994 | Cantanhéz - Canamine - Lala |
| LISC131195 | Combretaceae | *Combretum micranthum* G.Don | Amélia Moreira | 81 | 02/02/1994 | Cantanhéz - Catchamba Nalu |
| LISC131196 | Combretaceae | *Terminalia macroptera* Guill. & Perr. | M.A. Diniz & L.Catarino | 1756 | 17/11/1996 | Região de Tombali, Estação de Quebo (Coli) |
| LISC003062 | Connaraceae | *Cnestis ferruginea* Vahl ex DC. | L. Catarino & Q. Bancessi | 565 | 08/04/2000 | Região de Oio, setor de Bissorã. 12º 18' 17"N / 15º 32' 46"W |
| LISC131197 | Convolvulaceae | *Ipomoea aquatica*Forssk. | L. Catarino & A. Djaló | 276 | 06/12/1997 | Região de Quinara, Parque Natural das Lagoas de Cufada. 11º 45' 05"N / 15º 04' 26"W |
| LISC131198 | Cucurbitaceae | *Trichosanthes cucumerina* L. | J. Espirito Santo | 1234 | 15/03/1934 | Pessubé |
| LISC131199 | Cyperaceae | *Cyperus articulatus*L. | Amélia Moreira | 121 | 10/02/1994 | Cantanhéz - Sogobol |
| LISC091595 | Dilleniaceae | *Tetracera potatoria*Afzel. ex G.Don | Amélia Moreira | 250 | 16/06/1994 | Cantanhéz, Catchamba Nalu |
| LISC131200 | Dioscoreaceae | *Dioscorea bulbifera*L. | E.S. Martins & L. Catarino | 1336 | 20/11/1997 | Parque Natural das Lagoas de Cufada. 11º 39' 01" N / 15º 12' 09"W |
| LISC131201 | Dioscoreaceae | *Dioscorea hirtiflora*Benth. | L. Catarino & M.A. Djaló | 250 | 03/12/1997 | Região de Quinara. Parque Natural das Lagoas de Cufada. Buba Tumbo. 11º 38' 59"N / 15º 00' 56" W |
| LISC131202 | Dioscoreaceae | *Dioscorea mangenotiana* J.Miège | M. Adélia Diniz, I. Moreira & A. Moreira | 978 | 18/11/1994 | Iemberém. Tabanca, Catchamba. |
| LISC131203 | Ebenaceae | *Diospyros elliotii* (Hiern) F.White | E.S. Martins & L. Catarino | 1693 | 04/05/2001 | Quinara, Parque Natural das Lagoas de Cufada. 11º 42' 07"N / 14º 59' 32"W |
| LISC131204 | Ebenaceae | *Diospyros heudelotii* Hiern | E.S. Martins & L. Catarino | 1098 | 29/05/1996 | Parque Natural das Lagoas de Cufada. 11º 43' N / 15º 01' 43.3"W |
| LISC131205 | Fabaceae | *Cordyla pinnata*(A.Rich.) Milne-Redh. | E.S. Martins & L. Catarino | 1611 | 24/04/2001 | Quinara, Parque Natural das Lagoas de Cufada. 11º 43' 50" N / 14º 53' 55"W |
| LISC131206 | Fabaceae | *Dialium guineense*Willd. | M.A.Diniz, E.Gomes & O. Silva | 2531 | 27/03/1998 | Iemberém, Madina. 11º 13' 18" N / 15º 02' 28" W |
| LISC131207 | Fabaceae | *Parkia biglobosa*(Jacq.) R.Br. ex G.Don | M.Adélia Diniz & A.E. Gonçalves | 1790 | 15/04/1997 | Região de Cacheu, Setor de São Domingos. Elia. 12º 19' 22"N / 16º 23' 57"W |
| LISC131208 | Fabaceae | *Piliostigma thonningii* (Schumach.) Milne-Redh. | M.Adélia Diniz, A.E. Gonçalves & L. Catarino | 1338 | 27/10/1995 | Cantanhéz, Cabedú |
| LISC131209 | Fabaceae | *Pterocarpus santalinoides* L'Hér. ex DC. | E.S. Martins & L. Catarino | 1647 | 28/04/2001 | Quinara, Parque Natural das Lagoas de Cufada. 11º 43' 20" N / 15º 01' 41"W |
| LISC131210 | Fabaceae | *Senna alata*(L.) Roxb. | M.A. Diniz & L.Catarino | 1728 | 16/11/1996 | Região de Quinara, Parque Natural das Lagoas de Cufada. 11º 44' N / 14º 54' W |
| LISC131211 | Fabaceae | *Senna occidentalis*(L.) Link | M. Adélia Diniz, A. E. Gonçalves & L. Catarino | 996 | 17/10/1995 | Região de Cacheu, setor de São Domingos, seção de Varela, Bairro Lala. 12º 17' 23" N / 16º 34' 25"W |
| LISC116436 | Icacinaceae | *Icacina oliviformis*(Poir.) J.Raynal | M. Adélia Diniz & A.E. Gonçalves | 1923 | 19/04/1997 | Região de Cacheu, Setor de São Domingos, São Domingos. 12º 23' 51" N / 16º 12' 49"W |
| LISC131212 | Lamiaceae | *Platostoma africanum* P.Beauv. | M.L. Gonçalves, M.F.P. Basto, U. Candé, Q. Bancessi & F. Sané | 90 | 02/11/1988 | Provincia Leste, Região de Gabú, Setor de Pirada, Candjufa. |
| LISC131213 | Lamiaceae | *Vitex doniana*Sweet | E.S. Martins & I. Moreira | 983 | 28/05/1995 | Cantanhéz, Rio Cumbijâ, porto de Cadique |
| LISC131214 | Lamiaceae | *Vitex madiensis*Oliv. | Amélia Moreira | 262a | 02/07/1994 | Cantanhéz, Canamine, Lala |
| LISC131215 | Loganiaceae | *Strychnos spinosa*Lam. | M. Adélia Deniz & A.E. Gonçalves | 1957 | 21/04/1997 | Região de Cacheu, Setor de São Domingos, estrada de Varela para S. Domingos, Elia. |
| LISC102948 | Malvaceae | *Adansonia digitata* L. | Amélia Moreira | 254 | 21/06/1994 | Cantanhéz - Catchamba Nalu |
| LISC102954 | Malvaceae | *Bombax costatum*Pellegr. & Vuillet | J. Alves Pereira | 1193 | 06/01/1961 | Olossato, Madina |
| LISC102977 | Malvaceae | *Ceiba pentandra*(L.) Gaertn. | Amélia Moreira | 59 | 10/12/1993 | Iembrém. Caminho. Carreiro Cadique Iala/Cadique Nalu. |
| LISC103514 | Malvaceae | *Cola cordifolia*(Cav.) R.Br. | M. Adélia Diniz, A.E. Gonçalves & L. Catarino | 1010 | 17/10/1995 | Região de Cacheu, Setor de São Domingos, seção de Varela. Bairro Iale. 12º17' 42,6"N / 16º 34 ' 10.1"W |
| LISC106048 | Malvaceae | *Corchorus aestuans*L. | M. A. Diniz, M.F. Pinto Basto & S. Tadiconda | 2326 | 21/10/1997 | Arquipélago dos Bijagós. Ilha de Canhabaque, tabanca bine. |
| LISC102432 | Malvaceae | *Hibiscus cannabinus*L. | L. Catarino | 1493 | 18/03/2005 | Região de Tombali, setor de Quebo. Saré Amadi. 12º 31' 18"N / 14º 42' 24"W |
| LISC102550 | Malvaceae | *Hibiscus surattensis*L. | M. Paula Vidigal, M.F.P. Basto, S. Tadiconda & F. Cardoso | 279 | 10/12/1995 | Arquipélago dos Bijagós. Ilha de Canhabaque, Cudjuno. 11º 15' 24.2"N / 15º 39' 37'3"W |
| LISC103563 | Malvaceae | *Melochia corchorifolia*L. | L. Catarino & A. Djaló | 272 | 06/12/1997 | Região de Quinara, Parque Natural das Lagoas de Cufada. 11º 45' 05"N / 15º 04'26"W |
| LISC103644 | Malvaceae | *Sterculia tragacantha*Lindl. | Brigada de Estudos Florestais da Guiné | 117 | 07/01/1944 | Estrada de Teixeira Pinto |
| LISC131216 | Melastomataceae | *Dissotis grandiflora*(Sm.) Benth. | Amélia Moreira | 295 | 16/11/1994 | Cantanhéz - Canamine - Lala |
| LISC112923 | Meliaceae | *Carapa procera* DC | Adélia Diniz & A.E. Gonçalves | 1906 | 18/04/1997 | Região de Cacheu, Setor de São Domingos, Varela, Catão. 12º 17' 22" N / 16º 32' 17"W |
| LISC093712 | Menispermaceae | *Dioscoreophyllum cumminsii* (Stapf) Diels | Amélia Moreira | 281 | 28/10/1994 | Cantanéez, Catchamba Nalu |
| LISC093725 | Menispermaceae | *Triclisia patens*Oliv. | M.A. Diniz, E.Gomes & O. Silva | 2568 | 29/03/1998 | Iemberém, tabanca Farim. 11º 11' 53" N / 15º 4' 10" W |
| LISC131219 | Moraceae | *Ficus dicranostyla*Mildbr. | M.F.P. Basto; U. Candé; Q. Bancessi & Q. Correia | 183 | 13/06/1989 | Provincia do Norte, Região de Oio, Setor de Mansoa, Mambonco |
| LISC131217 | Moraceae | *Ficus sur*Forssk. | L. Catarino & Q. Bancessi | 408 | 02/04/2000 | Região de Gabú, Setor do Boé, Picada Contabane. Dandu. 11º 43' 46"N / 14º 12' 47"W |
| LISC131218 | Moraceae | *Treculia africana*Decne. ex Trécul | L. Catarino & Q. Bancessi | 941 | 10/05/2000 | Região de Tombali, setor de Bedanda. Cantanhéz. Cafatche. 11º 11' 05"N / 15º 06' 49"W |
| LISC131220 | Myristicaceae | *Pycnanthus angolensis* (Welw.) Warb. | E.S. Martins & I. Moreira | 881 | 25/05/1995 | Cantanhez, Floresta de Cambeque |
| LISC003070 | Myrtaceae | *Syzygium guineense*(Willd.) DC. | L. Catarino & Q. Bancessi | 1066 | 16/05/2000 | Região de Cacheu, setor de S. Domingos, entre S. Domingos e Suzana. 11º 23' 17"N / 16º 16' 33"W |
| LISC131221 | Nyctaginaceae | *Boerhavia erecta*L. | M. Paula Vidigal, M.F.P. Basto, Q. Bancessi & A. M. Embaló | 54 | 08/11/1991 | Provincia Leste, Região de Gabu, Sonaco, tabanca Nemataba |
| LISC093864 | Nymphaeaceae | *Nymphaea lotus*L. | L. Catarino & M.A. Djaló | 262 | 04/12/1997 | Região de Quinara. Parque Natural das Lagoas de Cufada. 11º 42' 54" N / 15º 01 40"W |
| LISC093888 | Nymphaeaceae | *Nymphaea micrantha*Guill. & Perr. | L. Catarino & M.A. Djaló | 263 | 05/12/1997 | Região de Quinara. Parque Natural das Lagoas de Cufada. 11º 42' 54" N / 15º 01 40"W |
| LISC110416 | Ochnaceae | *Lophira lanceolata*Tiegh. ex Keay | L. Catarino & Q. Bancessi | 377 | 01/04/2000 | Região de Tombali, Setor de Quebo. Picada Contabane - Boé. 11º 34' 21"N / 14º 38' 13"W |
| LISC115843 | Olacaceae | *Ximenia americana*L. | M.L. Gonçalves, M.F:P. Basto, U. Candé, Q. Bancessi & F. Sané | 67 | 01/11/1988 | Provincia de Leste, Região de Bafatá, Contuboel, campo de arroz de sequeiro da DEPA. |
| LISC131222 | Passifloraceae | *Smeathmannia laevigata*Sol. ex R.Br. | E.S.Martins & L. Catarino | 1680 | 03/05/2001 | Quinara, Parque Natural das Lagoas de Cufada, entre Buba Tumbo e a antinga Madina Chiripe. |
| LISC131223 | Passifloraceae | *Passiflora foetida*L. | L. Catarino & Q. Bancessi | 679 | 14/04/2000 | Região de Cacheu, setor de Cacheu. 12º 17' 25"N / 16º 05 05"W |
| LISC131224 | Pedaliaceae | *Sesamum radiatum*Thonn. ex Hornem. | F. Sané | 185 | 20/10/1988 | Cacheu, Bula, Quartel de Bassi. |
| LISC131225 | Phyllanthaceae | *Bridelia micrantha* (Hochst.) Baill. | J. Alves Pereira | 1950 | 16/05/1961 | Bafatá - Mato do Cão. |
| LISC131227 | Poaceae | *Cymbopogon caesius* (Hook. & Arn.) Stapf | E.S. Martins & L. Catarino | 1306 | 19/11/1997 | Parque Natural das Lagoas de Cufada, entes rio Fulacunda e Rio Grande de Buba. 11º 45' 33"N / 15º 10' 16"W |
| LISC131226 | Poaceae | *Digitaria longiflora*(Retz.) Pers. | M.A. Diniz, M.F. Pinto Basto & S.Tadiconda | 2376 | 23/10/1997 | Arquipélago dos Bijagós. Ilha de Canhabaque. Praia de Bine. 11º 13' N / 15º 46' 15"W |
| LISC122969 | Rhamnaceae | *Ziziphus mauritiana*Lam. | Espirito Santo | 3564 | 03/01/1955 | Gabú, entre Camalijam e Pirada |
| LISC131229 | Rhizophoraceae | *Rhizophora mangle* L. | E.S. Martins & M.Catarino | 1651 | 29/04/2001 | Quinara, Parque Natural das Lagoas de Cufada, rio Grande de Buba, Porto Sanindja. |
| LISC131228 | Rhizophoraceae | *Rhizophora x harrisonii* Leechm. | L. Catarino & Q. Bancessi | 665 | 14/04/2000 | Região de Cacheu, setor de Cacheu, porto de Cobiana. 12º 17' 12"N / 16º 05' 44"W |
| LISC131230 | Rubiaceae | *Macrosphyra longistyla*(DC.) Hook.f. ex Hiern | E.S. Martins & L. Catarino | 1498 | 27/11/1997 | Parque Natural das Lagoas de Cufada, entre Buba e Quebo. 11º 37' 21"N / 14º 54' 33"W |
| LISC131232 | Rubiaceae | *Nauclea latifolia*Sm. | M.A. Diniz & L.Catarino | 1558 | 12/11/1996 | Região de Quinara. Parque Natural das Lagoas de Cufada. Cantanha. 11º 43' N / 15º 01.5' W |
| LISC131231 | Rubiaceae | *Psychotria peduncularis* (Salisb.) Steyerm. | M.A. Diniz, E. Gomes & O. Silva | 2560 | 28/03/1998 | Iemberém, Cadique Maila. 11º 14' 32" N / 15º 7' 46" W |
| LISC126279 | Sapindaceae | *Allophylus africanus*P.Beauv. | E.S. Martins & L. Catarino | 1128 | 30/05/1996 | Parque Natural das Lagoas de Cufada. Estrada Buba - Fulacunda. 11º 41' 21" N / 15º 02' 33" W |
| LISC126343 | Sapindaceae | *Blighia sapida*K.D.Koenig | M. Adélia Diniz, & A. E. Gonçalves | 1845 | 16/04/1997 | Região de Cacheu, Setor de São Domingos, Varela, Bairro Iale. 12º 19' 22" N / 16º 34' 11" W |
| LISC126429 | Sapindaceae | *Lecaniodiscus cupanioides* Planch. ex Benth. | L. Catarino | 367 | 17/05/1998 | Região de Quinara. Parque Natural das Lagoas de Cufada. 11º 42' 30" N / 15º 05' 40"W |
| LISC126311 | Sapindaceae | *Lepisanthes senegalensis* (Poir.) Leenh. | M. Adélia Diniz & A.E. Gonçalves | 1946 | 19/04/1997 | Região de Cacheu, Setor de S. Domingos, estrada Suzana para Varela, na parte esquerda da estrada de Suzana para Varela. |
| LISC131233 | Solanaceae | *Physalis angulata*L. | Amélia Moreira | 274 | 30/09/1994 | Cantanhéz - Catchamba Nalu |
| LISC124839 | Vitaceae | *Cissus populnea*Guill. & Perr. | E. S. Martins & L. Catarino | 1321 | 19/11/1997 | Parque Natural das Lagoas de Cufada, entre Fulacunda e Uaná Porto. 11º 47' 15"N / 15º 08' 53"W |
| LISC131234 | Zingiberaceae | *Aframomum alboviolaceum* (Ridl.) K.Schum. | Amélia Moreira | 299 | 25/11/1994 | Cantanhéz - Canamine - Lala |
| LISC131239 | Zingiberaceae | *Aframomum cereum* (Hook.f.) K.Schum. (= *A. sceptrum* (Oliv. & D.Hanb.) K.Schum.) | Espirito Santo | 2261 | 19/06/1946 | Entre Sonaco e Gabú |
| LISC131238 | Zingiberaceae | *Aframomum rostratum* K.Schum. | Martins, Moreira, Bancessi & Candé | 622 | 08/12/1992 | Região Sul, entre Quebo e Caboxanque, proximo do cruzamento para Iemberém. |

**Supplementary Table S4.** Checklist of Guinea-Bissau’s wild edible plants, with references to plant parts used and types of edible use. The 13 species highlighted in bold are used as "famine food" when preferred foodstuff is not available, namely during food shortages.

| **Family** | **Species** | **Plant parts used** | **Type of edible use** |
| --- | --- | --- | --- |
| Acanthaceae | ***Avicennia germinans* (L.) L.** | **Fruit** | **Food*** |
|  | *Nelsonia canescens* (Lam.) Spreng. | Leaves | Food, Spice |
| Aizoaceae | *Sesuvium portulacastrum* (L.) L. | Leaves | Food |
| Amaranthaceae | *Alternanthera nodiflora* R.Br. | Leaves | Food |
|  | *Amaranthus cruentus* L. | Leaves | Food |
|  | *Amaranthus spinosus* L. | Leaves | Food |
|  | *Amaranthus viridis* L. | Leaves | Food |
|  | *Celosia argentea* L. | Leaves | Food, Drink |
|  | *Gomphrena vermicularis* L. | Leaves | Food |
| Anacardiaceae | *Lannea acida* A.Rich. | Fruit | Food |
|  | *Lannea nigritana* (Scott Elliot) Keay | Fruit | Food |
|  | *Lannea velutina* A.Rich. | Fruit | Food |
|  | *Pseudospondias microcarpa* (A.Rich.) Engl. | Fruit | Food |
|  | *Sclerocarya birrea* (A.Rich.) Hochst. | Fruit | Food |
|  | *Sorindeia juglandifolia* (A.Rich.) Planch. ex Oliv. | Fruit | Food |
|  | *Spondias mombin* L. | Fruit | Food, Drink |
| Anisophylleaceae | *Anisophyllea laurina* R.Br. ex Sabine | Fruit | Food |
| Annonaceae | *Annona glabra* L. | Fruit | Food |
|  | *Annona glauca* Schumach. & Thonn. | Fruit | Food |
|  | *Annona senegalensis* Pers. | Fruit | Food |
|  | *Uvaria chamae* P.Beauv. | Fruit | Food |
|  | *Xylopia aethiopica* (Dunal) A.Rich. | Fruit, Roots | Drink, Spice |
| Apocynaceae | *Landolphia dulcis* (Sabine ex G.Don) Pichon | Fruit | Food |
|  | *Landolphia heudelotii* A.DC. | Fruit | Food, Drink, Spice |
|  | *Landolphia hirsute* (Hua) Pichon | Fruit | Food |
|  | *Landolphia incerta* (K.Schum.) J.G.M.Pers. | Fruit | Food |
|  | *Landolphia owariensis* P.Beauv. | Fruit | Food, Drink |
|  | *Leptadenia lanceolata* (Poir.) Goyder | Leaves, Flowers | Food, Drink |
|  | *Mondia whitei* (Hook.f.) Skeels | Fruit, Roots | Food, Drink, Sweetener |
|  | *Saba comorensis* (Bojer ex A.DC.) Pichon | Fruit | Food |
|  | *Saba senegalensis* (A.DC.) Pichon | Fruit | Food, Drink |
|  | *Tacazzea apiculata* Oliv. | Leaves | Food |
|  | *Voacanga africana* Stapf | Fruit | Food |
| Araliaceae | *Cussonia arborea* Hochst. ex A.Rich. | Fruit | Food |
| Arecaceae | ***Borassus aethiopum* Mart.** | **Fruit, Seeds, Sprouts** | **Food*, Drink** |
|  | ***Borassus akeassii* Bayton, Ouédr. & Guinko** | **Fruit, Seeds, Sprouts** | **Food*, Drink** |
|  | ***Elaeis guineensis* Jacq.** | **Fruit, Seeds, Sap** | **Food*, Sprouts, Drink, Edible oil** |
|  | *Hyphaene thebaica* (L.) Mart. | Fruit | Food, Drink |
|  | *Phoenix reclinata* Jacq. | Fruit | Food, Drink |
|  | *Raphia palma-pinus* (Gaertn.) Hutch. | Fruit | Food, Drink |
|  | *Raphia sudanica* A.Chev. | Fruit | Food |
| Celastraceae | *Salacia senegalensis* (Lam.) DC. | Fruit | Food |
| Chrysobalanaceae | *Chrysobalanus icaco* L. | Fruit | Food |
|  | *Neocarya macrophylla* (Sabine) Prance ex F.White | Fruit, Seeds | Food |
|  | *Parinari curatellifolia* Planch. ex Benth. | Fruit, Seeds | Food |
|  | *Parinari excelsa* Sabine | Fruit, Seeds | Food, Drink |
| Combretaceae | *Combretum micranthum* G.Don | Leaves | Drink |
|  | *Terminalia macroptera* Guill. & Perr. | Leaves | Drink |
| Connaraceae | *Cnestis ferruginea* Vahl ex DC. | Fruit | Food |
| Convolvulaceae | *Ipomoea aquatica* Forssk. | Roots | Food |
| Cucurbitaceae | *Trichosanthes cucumerina* L. | Fruit | Food |
| Cyperaceae | *Cyperus articulatus* L. | Roots | Food |
| Dilleniaceae | *Tetracera potatoria* Afzel. ex G.Don | Leaves | Food |
| Dioscoreaceae | ***Dioscorea bulbifera* L.** | **Roots** | **Food*** |
|  | ***Dioscorea hirtiflora* Benth.** | **Roots** | **Food*** |
|  | ***Dioscorea mangenotiana* J.Miège** | **Roots** | **Food*** |
| Ebenaceae | *Diospyros elliotii* (Hiern) F.White | Fruit | Food |
|  | *Diospyros heudelotii* Hiern | Fruit | Food |
| Fabaceae | *Cordyla pinnata* (A.Rich.) Milne-Redh. | Fruit | Food |
|  | *Dialium guineense* Willd. | Fruit, Leaves | Food, Drink |
|  | ***Parkia biglobosa* (Jacq.) R.Br. ex G.Don** | **Fruit, Seeds** | **Food*, Drink, Spice** |
|  | *Piliostigma thonningii* (Schumach.) Milne-Redh. | Leaves | Drink |
|  | *Pterocarpus santalinoides* L'Hér. ex DC. | Seeds | Food |
|  | *Senna alata* (L.) Roxb. | Seeds | Drink |
|  | *Senna occidentalis* (L.) Link | Leaves, Seeds | Drink |
| Icacinaceae | ***Icacina oliviformis* (Poir.) J.Raynal** | **Roots, Fruit** | **Food*** |
| Lamiaceae | *Platostoma africanum* P.Beauv. | Leaves | Spice |
|  | *Vitex doniana* Sweet | Fruit | Food |
|  | *Vitex madiensis* Oliv. | Fruit | Food |
| Loganiaceae | *Strychnos spinosa* Lam. | Fruit | Food |
| Malvaceae | *Adansonia digitata* L. | Fruit, Fl, Leaves | Food, Drink |
|  | *Bombax costatum* Pellegr. & Vuillet | Flowers, Leaves | Food |
|  | *Ceiba pentandra* (L.) Gaertn. | Leaves, Flowers | Food |
|  | *Cola cordifolia* (Cav.) R.Br. | Fruit | Food |
|  | *Corchorus aestuans* L. | Leaves | Food |
|  | ***Hibiscus cannabinus* L.** | **Leaves** | **Food*** |
|  | ***Hibiscus surattensis* L.** | **Leaves** | **Food*** |
|  | *Melochia corchorifolia* L. | Leaves | Food |
|  | *Sterculia tragacantha* Lindl. | Leaves | Food |
| Melastomataceae | *Dissotis grandiflora* (Sm.) Benth. | Roots | Swetener |
| Meliaceae | *Carapa procera* DC | Seeds | Food |
| Menispermaceae | *Dioscoreophyllum volkensii* Engl. | Fruit | Food |
| Menispermaceae | *Triclisia patens* Oliv. | Fruit | Food |
| Moraceae | *Ficus dicranostyla* Mildbr. | Leaves | Food |
|  | *Ficus sur* Forssk. | Fruit | Food |
|  | *Treculia africana* Decne. ex Trécul | Seeds | Food |
| Myristicaceae | *Pycnanthus angolensis* (Welw.) Warb. | Leaves | Food |
| Myrtaceae | *Syzygium guineense* (Willd.) DC. | Fruit | Food |
| Nyctaginaceae | *Boerhavia erecta* L. | Leaves | Food |
| Nymphaeaceae | *Nymphaea lotus* L. | Roots | Food |
|  | *Nymphaea micrantha* Guill. & Perr. | Roots | Food |
| Ochnaceae | *Lophira lanceolata* Tiegh. ex Keay | Fruit | Edible oil |
| Olacaceae | *Ximenia americana* L. | Fruit | Food |
| Passifloraceae | *Smeathmannia laevigata* Sol. ex R.Br. | Fruit | Food |
|  | *Passiflora foetida* L. | Fruit | Food |
| Pedaliaceae | *Sesamum radiatum* Thonn. ex Hornem. | Leaves, Flowers | Food |
| Phyllanthaceae | *Bridelia micrantha* (Hochst.) Baill. | Fruit, Bark | Food, Spice |
| Poaceae | *Cymbopogon caesius* (Hook. & Arn.) Stapf | Leaves | Spice |
|  | ***Digitaria longiflora* (Retz.) Pers.** | **Seeds** | **Food*** |
| Rhamnaceae | *Ziziphus mauritiana* Lam. | Fruit | Food |
| Rhizophoraceae | *Rhizophora mangle* L. | Roots | Food |
|  | *Rhizophora x harrisonii* Leechm. | Roots | Food |
| Rubiaceae | *Macrosphyra longistyla* (DC.) Hook.f. ex Hiern | Fruit | Food |
|  | *Nauclea latifolia* Sm. | Fruit | Food |
|  | *Psychotria peduncularis* (Salisb.) Steyerm. | Fruit | Food |
| Sapindaceae | *Allophylus africanus* P.Beauv. | Fruit | Food |
|  | *Blighia sapida* K.D.Koenig | Fruit | Food |
|  | *Lecaniodiscus cupanioides* Planch. ex Benth. | Fruit | Food |
|  | *Lepisanthes senegalensis* (Poir.) Leenh. | Fruit | Food |
| Solanaceae | *Physalis angulata* L. | Fruit | Food |
| Vitaceae | ***Cissus populnea* Guill. & Perr.** | **Fruit** | **Food*** |
| Zingiberaceae | *Aframomum alboviolaceum* (Ridl.) K.Schum. | Fruit | Food |
|  | *Aframomum cereum* (Hook.f.) K.Schum. | Fruit | Food |
|  | *Aframomum melegueta* K.Schum. | Fruit | Food |
|  | *Aframomum rostratum* K.Schum. | Fruit | Food |
